# Faricimab 6 mg versus aflibercept 8 mg in diabetic macular oedema: an *in silico* study of anchored efficacy and common-protocol treatment burden

**DOI:** 10.3389/fphar.2026.1938908

**Published:** 2026-10-13

**Authors:** Georgios D. Panos

**Affiliations:** 1 Department of Ophthalmology, AHEPA University Hospital, School of Medicine, Aristotle University of Thessaloniki, Thessaloniki, Greece; 2 Division of Ophthalmology and Visual Sciences, School of Medicine, University of Nottingham, Nottingham, United Kingdom

**Keywords:** aflibercept 8 mg, diabetic macular oedema, durability, faricimab, indirect treatment comparison, microsimulation, treatment burden

## Abstract

**Background:**

No prospective randomised head-to-head trial has compared faricimab 6 mg with aflibercept 8 mg for diabetic macular oedema (DMO). Their pivotal programmes used materially different dosing architectures, precluding interpretation of observed interval distributions as drug properties.

**Methods:**

We separated an anchored Bayesian aggregate-data comparison of year-1 and year-2 best-corrected visual acuity (BCVA) and retinal-thickness differences from an individual-level, discrete-time burden microsimulation. The latter applied identical three-dose loading and Q4W–Q16W rules, conditional on transported durability evidence. Protocol standardisation applied only to burden; dosing history did not generate efficacy. Inputs represented mixed pivotal-trial populations. Structural, prior, composite leave-out, qualification and calibration analyses were performed.

**Results:**

The estimated BCVA difference between treatments was small and compatible with no treatment difference: faricimab minus aflibercept 8 mg was +1.22 letters at year 1 (95% uncertainty interval [UI], −0.48 to +2.86) and +0.41 at year 2 (−1.43 to +2.27). Retinal-thickness-reduction differences were +21.93 μm at year 1 (+1.70 to +42.52; P[Δ > 0] = 98.3%) and +20.53 μm at year 2 (−1.08 to +42.24); the year-2 interval included zero, and estimates varied across structures. Under the common simulated treat-and-extend regimen, aflibercept 8 mg was projected to require fewer injections than faricimab (7.29 vs. 8.01 by week 52 and 10.93 vs. 12.60 by week 104). A greater proportion of simulated eyes receiving aflibercept 8 mg than faricimab reached 16-week dosing intervals (80.2% vs. 50.9% at week 52 and 77.7% vs. 60.4% at week 104). Aflibercept 8 mg showed a lower estimated injection burden, although the adjusted range included no difference between treatments.

**Conclusion:**

The estimated BCVA differences were small and compatible with no treatment difference. Faricimab showed greater anatomical improvement at year 1, while the year-2 estimate included no difference. Aflibercept 8 mg showed a lower simulated injection burden, although the adjusted range included no difference. Neither treatment demonstrated clinical superiority.

## Introduction

Diabetic macular oedema (DMO) is a major cause of visual impairment in people with diabetes and frequently requires prolonged treatment to preserve or improve vision (Browning et al., 2018). Intravitreal inhibition of vascular endothelial growth factor (VEGF) is standard care for centre-involving DMO, but the benefits achieved in randomised trials depend on repeated injections, monitoring and timely retreatment (Schmidt-Erfurth et al., 2017). Maintaining this intensity can be difficult and creates a need for treatments that sustain disease control while reducing treatment frequency (Nakao et al., 2024).

Faricimab is a bispecific antibody that inhibits VEGF-A and angiopoietin-2. In YOSEMITE and RHINE, faricimab every 8 weeks or according to a personalised treatment-interval regimen achieved BCVA improvements non-inferior to aflibercept 2 mg every 8 weeks, with greater central-subfield-thickness reductions in several analyses (Wong et al., 2024; Wykoff et al., 2022). Aflibercept 8 mg was developed to increase molar dose and extend VEGF suppression. In PHOTON, aflibercept 8 mg every 12 or 16 weeks after initial monthly dosing achieved BCVA gains non-inferior to aflibercept 2 mg every 8 weeks, with outcomes maintained through 96 weeks (Brown et al., 2024; Do et al., 2026).

No prospective randomised head-to-head trial has compared faricimab 6 mg with aflibercept 8 mg. Two small retrospective cohorts reported short-term direct comparisons, but neither estimated long-term outcomes under a common treat-and-extend protocol (Maccauro et al., 2025; Menna et al., 2026). An anchored network meta-analysis suggested no material BCVA difference and fewer observed injections with aflibercept 8 mg than with faricimab personalised treatment intervals (Friedman et al., 2025; 2026). Interpretation remains difficult because pivotal programmes differed in loading, interval assignment, extension, maintenance and shortening. YOSEMITE/RHINE intervals were achieved through response-dependent personalised extension, whereas PHOTON Q12W or Q16W regimens were assigned and then maintained or modified (Brown et al., 2024; Do et al., 2026; Wong et al., 2024; Wykoff et al., 2022). Observed interval distributions therefore combine treatment response with protocol design.

The aim of this study was to estimate the relative functional and anatomical efficacy of faricimab 6 mg and aflibercept 8 mg through an anchored aggregate-data indirect comparison and, separately, to explore treatment burden under a common fully adherent trial-like algorithm. Its distinctive contribution was to estimate comparative burden after both agents were assigned the same three-dose loading phase, Q4W–Q16W interval limits and extension/shortening rules. Efficacy outcomes and treatment burden were evaluated using separate analytical components.

## Methods

### Study design and estimands

The study comprised two analytically separate components (Figure 1). Component A was a Bayesian aggregate-data indirect comparison of functional and anatomical efficacy, anchored through the aflibercept 2 mg groups within each pivotal trial. Component B was an individual-level, discrete-time virtual-patient microsimulation of treatment burden under a common algorithm, conditional on transport of durability information from the source regimens. The components shared published evidence sources but did not exchange simulated outcomes, and injection history had no effect on the efficacy estimates.

**FIGURE 1:**
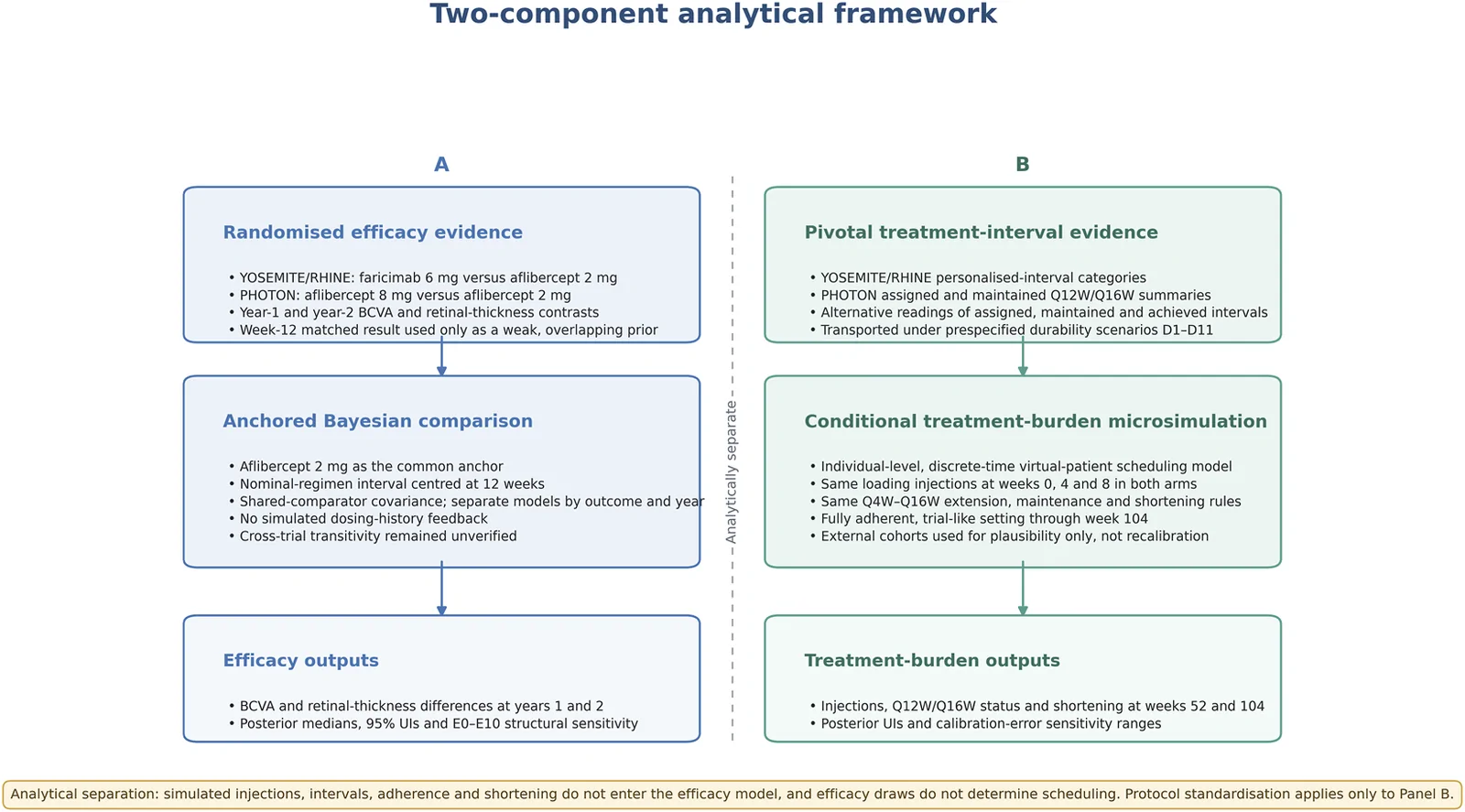
Two-component analytical framework. Efficacy was estimated through an anchored aggregate-data indirect comparison and was not generated by the simulated dosing protocol. Treatment burden was explored separately using an individual-level, discrete-time microsimulation conditional on transported durability assumptions. Protocol standardisation applies only to the burden component. **(A)** Aggregate efficacy ‐ not protocol-standardised **(B)** Common-algorithm burden ‐ protocol-standardised.

For efficacy, the estimand was the faricimab-minus-aflibercept 8 mg difference in mean BCVA change or mean central-retinal-thickness reduction at year 1 and year 2, evaluated at the model reference value corresponding to a nominal 12-week regimen label. This meta-regression adjustment did not recreate a common dosing history or protocol-standardise efficacy. The estimate is an anchored model-based contrast, not a causal treatment effect, because cross-trial transitivity could not be established. For treatment burden, the estimands were conditional differences in cumulative injections, Q12W-or-longer status, Q16W status and interval shortening by weeks 52 and 104 under the common simulated schedule.

### Evidence identification, sources and extraction

Evidence was identified through a targeted, non-systematic search of MEDLINE/PubMed and ClinicalTrials.gov, supplemented by journal and trial-registry records, conference materials and backward citation searching. Searches were updated to 30 June 2026 and combined terms for DMO, faricimab, aflibercept 8 mg, YOSEMITE, RHINE, PHOTON, visual acuity, retinal thickness, treatment interval, injections and safety. Eligible model-input sources reported aggregate efficacy, anatomical, interval, injection or safety outcomes for the pivotal regimens or prespecified external-plausibility cohorts. Two short-term retrospective direct comparisons were retained for context but excluded from model inputs, calibration and validation because neither evaluated long-term outcomes under a common protocol (Maccauro et al., 2025; Menna et al., 2026). Sources and analytical roles are summarised in Table 1; search terms and selection rules are provided in Supplementary Appendix S3.

**TABLE 1:** Evidence sources and analytical roles.

Source	Design/population	Relevant regimen	Extracted outcomes	Role
YOSEMITE/RHINE	Randomised phase 3; mixed prior-treatment population	Faricimab 6 mg Q8W/PTI; aflibercept 2 mg Q8W	BCVA, CST, injections, interval distributions, safety	Anchored faricimab efficacy contrasts; transported durability evidence; qualification; descriptive safety
PHOTON	Randomised phase 2/3; mixed prior-treatment population	Aflibercept 8 mg Q12W/Q16W; aflibercept 2 mg Q8W	BCVA, CRT, injections, assigned/maintained intervals, safety	Anchored aflibercept 8 mg efficacy contrasts; transported durability evidence; qualification; descriptive safety
Published week-12 matching-adjusted indirect comparison	YOSEMITE/RHINE reweighted to PHOTON aggregate baseline	Three Q4W injections before week-12 outcome	BCVA and CST contrasts; matching variables; effective sample size	Weak prior only; no matching performed in this study
FARWIDE-DMO	Retrospective UK study	Routine-practice faricimab	12-month injections	External plausibility only
Swiss Retina Research Network	Multicentre observational study	Routine-practice aflibercept 8 mg	12-month injections	External plausibility only
Barbosa et al.	Retrospective, predominantly previously treated	Routine-practice aflibercept 8 mg	Ability to maintain ≥7-week interval	External plausibility only
Maccauro et al.; Menna et al.	Small retrospective, non-randomised direct comparisons	Short-term faricimab 6 mg and aflibercept 8 mg regimens	Short-term BCVA and retinal-thickness outcomes; retinal sensitivity in Menna et al.	Context only; not model inputs, calibration targets or independent validation

BCVA, best-corrected visual acuity; CRT, central retinal thickness; CST, central subfield thickness; NMA, network meta-analysis; PTI, personalised treatment interval. Real-world sources did not inform estimation or calibration.

The principal randomised evidence sources were YOSEMITE and RHINE for faricimab and PHOTON for aflibercept 8 mg (Brown et al., 2024; Do et al., 2026; Wong et al., 2024; Wykoff et al., 2022). Each included an aflibercept 2 mg comparator. Extracted measures of uncertainty were reported standard errors, confidence or credible intervals, denominators, event counts and, where required, standard errors reconstructed from interval widths using the stated distributional approximation. Every value, transformation and source location is reported in Supplementary Tables S2–S4.

A published week-12 matching-adjusted indirect comparison supplied weak prior information after three every-4-week injections (Lim et al., 2026). It reweighted YOSEMITE and RHINE individual data to PHOTON aggregate characteristics using age, baseline BCVA, central subfield thickness, prior anti-VEGF exposure and Diabetic Retinopathy Severity Scale categories; effective sample sizes were 615.56 and 609.85 (rounded to 616 and 610). Because it reused pivotal-programme participants, it was treated as overlapping empirical prior information rather than independent evidence.

Baseline comparability was assessed using the aflibercept 2 mg groups (Table 2; Supplementary Table S5). YOSEMITE/RHINE reported central subfield thickness from the internal limiting membrane to Bruch’s membrane using Spectralis, Cirrus or Topcon spectral-domain OCT; PHOTON reported central retinal thickness from the internal limiting membrane to the retinal pigment epithelium. Source terminology was retained, and anatomical contrasts were interpreted cautiously rather than assumed interchangeable (Brown et al., 2024; Do et al., 2026; Wong et al., 2024; Wykoff et al., 2022).

**TABLE 2:** Cross-trial comparability of the aflibercept 2 mg common-comparator arms.

Characteristic	YOSEMITE	RHINE	PHOTON	Assessment
Patients	312	315	167	Different common-arm precision
Age, years, mean (SD)	62.2 (9.6)	62.3 (10.1)	63.0 (9.8)	Broadly similar
Male, %	57	59	55	Broadly similar
HbA1c, %, mean (SD)	7.6 (1.1)	7.7 (1.2)	8.1 (1.5)	Higher and more variable in PHOTON
BCVA, letters, mean (SD)	62.2 (9.5)	62.1 (9.4)	61.5 (11.2)	Broadly similar mean; wider SD in PHOTON
Retinal thickness, µm, mean (SD)	CST 484.5 (131.1)	CST 477.3 (129.4)	CRT 457.2 (144.0)	Different terminology/boundaries
Treatment-naïve, %	78	79	56	Material imbalance; estimand changed to mixed population
DMO duration, months, mean (SD)	17.5 (27.6)	20.3 (37.1)	Not reported	Transitivity cannot be checked
Diabetes duration, years, mean (SD)	Not reported	Not reported	15.9 (10.0)	Not jointly comparable
DRSS: Mild/moderate, %	58	57	63	Categories harmonised only approximately
DRSS: Moderately severe/severe, %	33	33	23	Potential imbalance
DRSS: PDR, %	6	6	8	Small difference
Region, %	USA/Canada 54; Asia 6; other 40	USA/Canada 35; Asia 8; other 57	Exact distribution not reported	Regional exchangeability unverified
Sites/countries	179/16	174/24	138/7	Different geographic composition
OCT platform	Spectralis, Cirrus or Topcon SD-OCT	Spectralis, Cirrus or Topcon SD-OCT	SD-OCT; distribution not reported	Platform distribution incompletely comparable
Segmentation	ILM to Bruch’s membrane (CST)	ILM to Bruch’s membrane (CST)	ILM to RPE (CRT)	Anatomical measures not interchangeable
Assessment schedule	Monitored Q4W; year-1 average weeks 48/52/56	Monitored Q4W; year-1 average weeks 48/52/56	Monitored Q4W; week 48	Landmarks differ
Year-1 BCVA gain, letters	10.9	10.3	8.7	Observed common-arm response differs
Year-1 thickness reduction, µm	170.3 CST	170.1 CST	164.8 CRT	Definition and landmark differ

Values refer to the aflibercept 2 mg groups. DMO, diabetic macular oedema; DRSS, Diabetic Retinopathy Severity Scale; ILM, internal limiting membrane; PDR, proliferative diabetic retinopathy; RPE, retinal pigment epithelium; SD-OCT, spectral-domain optical coherence tomography. Broad similarity on selected means does not establish transitivity; unmeasured effect modification remains possible.

### Population

The target population reflected the mixed populations enrolled in the pivotal trials rather than exclusively treatment-naïve patients. In the aflibercept 2 mg groups, 78%, 79% and 56% of participants in YOSEMITE, RHINE and PHOTON, respectively, were treatment-naïve (Brown et al., 2024; Do et al., 2026; Wong et al., 2024; Wykoff et al., 2022). Because outcomes stratified by previous anti-VEGF treatment were not consistently available, treatment history could not be adjusted for; therefore, the findings apply to a mixed pivotal-trial population of treatment-naïve and previously treated patients.

### Anchored efficacy model

Separate models were fitted for BCVA and retinal-thickness reduction at year 1 and year 2. Each included four faricimab-versus-aflibercept 2 mg contrasts from YOSEMITE/RHINE and two aflibercept 8 mg-versus-aflibercept 2 mg contrasts from PHOTON. The design matrix contained an intercept, a faricimab indicator and nominal regimen interval centred at 12 weeks. Contrasts sharing a comparator were assigned sampling correlation 0.50 in the reference analysis. Residual heterogeneity was integrated over a prespecified grid.

The published week-12 matching-adjusted comparison supplied prior means of +0.8 letters and +19.0 µm. For the year-1 model, standard deviations approximated from the corresponding week-12 95% credible intervals were multiplied by two to downweight the overlapping information; year-2 prior standard deviations were specified separately as 1.5 letters and 20 μm. E1 replaced this with weak zero-centred priors with standard deviations of 5 letters and 50 μm; E2 used sceptical zero-centred priors with standard deviations of 1 letter and 10 µm. Other analyses omitted the interval covariate, combined that omission with removal of the week-12 prior, allowed treatment-specific interval slopes and used fixed effects. E7–E10 were composite stress tests that omitted the nominal-interval covariate and week-12 prior and additionally omitted YOSEMITE, RHINE, PHOTON Q12W or PHOTON Q16W, respectively (Supplementary Table S1; Supplementary Figure S2). A ±3-letter region was descriptive, not a prespecified equivalence margin.

### Conditional treatment-burden microsimulation

Treatment burden was simulated using an individual-level, discrete-time virtual-patient microsimulation with a weekly event clock. Both treatments received loading injections at weeks 0, 4 and 8. At week 12, a pre-dose assessment determined the next interval and the fourth injection was given. Thus, the primary regimen contained three loading injections; a separate four-loading-injection scenario was retained as a sensitivity analysis.

Intervals were Q4W, Q8W, Q12W or Q16W and changed in 4-week increments. At each attended treatment visit, the current interval was compared with a latent sustainable interval generated from drug-, time- and patient-specific durability terms plus visit noise. The algorithm extended, maintained or shortened the next interval accordingly. This latent state represented the scheduling consequence of disease activity; BCVA, retinal thickness and fluid were not simulated as longitudinal observed outcomes and did not feed back into efficacy.

Durability at weeks 52 and 96 was represented by one-, two- or three-component latent structures under alternative readings of assigned, maintained and achieved interval evidence. For the two-component reference structure, the mixing proportion was interpolated on the logit scale, component means on the natural scale and residual variability on the log scale between weeks 52 and 96; year-1 parameters applied through week 52 and year-2 parameters from week 96 onwards. A persistent patient rank induced within-patient correlation without imposing an immutable subgroup: changing mixture weights allowed membership relative to component thresholds to change over time.

The reported-evidence scenario (D1) used source definitions most directly but was not interpreted as an intrinsic drug property. Alternative scenarios varied mixture components, category thresholds, visit noise, inclusion and interpretation of assigned or maintained intervals, and evidence discounting; the null scenario imposed equal durability (Supplementary Table S6). Primary adherence was 100%, corresponding to a fully adherent trial-like setting.

### Qualification, calibration and external plausibility

Before interpreting the between-treatment comparisons, the simulator was assessed against 17 injection-count and dosing-interval targets from the original trial regimens. Thirteen targets were classified as critical. Four PHOTON extended-interval targets were exploratory: two assessed intervals of at least 20 weeks and two assessed assigned 24-week intervals. Tolerances were one injection for injection-count targets, 10 percentage points for critical interval targets and 12 percentage points for the four exploratory extended-interval targets.

Because qualification tolerances and observed replication errors were similar in scale to some comparative effects, replication error was carried into burden sensitivity ranges. For each metric, the empirical bound was the sum of the maximum absolute original-regimen replication errors across treatments, and the nominal uncertainty interval was expanded by this bound. These deterministic calibration-error-expanded ranges are not posterior uncertainty intervals. A more conservative expansion using the engineering tolerance is reported in Supplementary Table S7.

FARWIDE-DMO, the Swiss Retina Research Network report and Barbosa et al. were used only for external plausibility (Barbosa et al., 2026; Peto et al., 2025; Spindler et al., 2026). They did not inform estimation or recalibration. The simulation assumed full adherence to protocol-defined visits and did not model missed visits or clinician-directed scheduling.

### Outcomes, safety and statistical reporting

Efficacy outcomes were mean BCVA change and mean retinal-thickness reduction. Model-derived responder proportions were removed because aggregate means did not identify individual response distributions. No individual baseline efficacy distribution or BCVA/thickness bound enters either revised estimand. Burden outcomes were cumulative injections, Q12W-or-longer status, Q16W status, any shortening, inability to maintain at least 7 weeks and active-treatment-visit fraction. DRSS was not included because comparable treatment-specific data on changes over time were unavailable. Retinal fluid was not assessed separately because the model estimated dosing intervals rather than changes in OCT findings.

Safety was outside the efficacy and scheduling models. A descriptive, non-comparative summary of intraocular inflammation, retinal vasculitis, endophthalmitis, intraocular pressure, Antiplatelet Trialists’ Collaboration arterial thromboembolic events, deaths and discontinuations was extracted from the source programmes. Differences in populations, exposure windows and ascertainment precluded comparative pooling.

Posterior medians and 95% uncertainty intervals are reported. Posterior probabilities are stated only where their event is defined. The virtual sample size was selected for numerical precision and does not create empirical observations.

### Computation and quality control

The final analysis used R 4.6.1, 4,000 reference efficacy draws, 3,000 draws for each efficacy structural case, 500 outer durability draws per scenario and 1,500 virtual patients per treatment arm. It completed without recorded warnings. Source values were checked twice by the author. Quality control comprised automated tests, manifest and checksum verification, exact reproduction of the submitted efficacy summaries and comparison of quick and full Monte Carlo runs.

## Results

### Cross-trial comparability

The aflibercept 2 mg arms were broadly similar in age, sex, baseline BCVA and retinal thickness, but important differences remained (Table 2; Supplementary Table S5). Mean HbA1c was 7.6%, 7.7% and 8.1% in YOSEMITE, RHINE and PHOTON, and treatment-naïve proportions were 78%, 79% and 56%. DMO duration was reported for YOSEMITE/RHINE but not PHOTON, whereas diabetes duration was reported for PHOTON. OCT boundaries differed. Year-1 aflibercept 2 mg BCVA gains were 10.9, 10.3 and 8.7 letters, and thickness reductions were 170.3, 170.1 and 164.8 µm, at non-identical landmarks and with different anatomical definitions (Brown et al., 2024; Do et al., 2026; Wong et al., 2024; Wykoff et al., 2022). These observations do not establish exchangeability; cross-trial transitivity remained unverified.

### Anchored efficacy comparison

At year 1, the estimated faricimab-minus-aflibercept 8 mg BCVA difference was +1.22 ETDRS letters (95% UI, −0.48 to +2.86; P[Δ > 0] = 92.1%). At year 2, it was +0.41 letters (−1.43 to +2.27; P[Δ > 0] = 67.4%). The estimated BCVA differences were small, with both uncertainty intervals including no treatment difference. Across all sensitivity analyses, estimated BCVA differences were small, and all 95% uncertainty intervals included no treatment difference for year 1 and 2 (Table 3; Figure 2A; Supplementary Table S1; Supplementary Figure S2).

**TABLE 3:** Anchored efficacy estimates and conditional model-predicted treatment burden.

Component	Outcome	Time	Faricimab 6 mg	Aflibercept 8 mg	Difference, faricimab − aflibercept 8 mg (95% UI)	Calibration-error-expanded sensitivity range
Anchored efficacy	BCVA difference, letters	Year 1	—	—	+1.22 (−0.48 to +2.86)	—
Anchored efficacy	BCVA difference, letters	Year 2	—	—	+0.41 (−1.43 to +2.27)	—
Anchored efficacy	Difference in thickness reduction, µm	Year 1	—	—	+21.93 (+1.70 to +42.52)	—
Anchored efficacy	Difference in thickness reduction, µm	Year 2	—	—	+20.53 (−1.08 to +42.24)	—
Burden D1	Cumulative injections	Week 52	8.01 (7.70–8.43)	7.29 (7.13–7.58)	+0.71 (+0.33 to +1.17)	−0.93 to +2.42
Burden D1	Cumulative injections	Week 104	12.60 (11.96–13.40)	10.93 (10.60–11.48)	+1.66 (+0.84 to +2.47)	−0.41 to +3.73
Burden D1	At Q12W or longer, %	Week 52	73.4 (64.9–80.9)	90.7 (85.1–95.2)	−17.2 pp (−27.0 to −8.4)	−30.4 to −5.0 pp
Burden D1	At Q12W or longer, %	Week 104	79.0 (72.2–86.2)	89.2 (82.1–93.7)	−10.1 pp (−18.8 to +0.1)	−22.3 to +3.6 pp
Burden D1	At Q16W, %	Week 52	50.9 (42.2–60.0)	80.2 (71.4–88.4)	−28.7 pp (−40.8 to −17.0)	−50.7 to −7.2 pp
Burden D1	At Q16W, %	Week 104	60.4 (52.6–69.6)	77.7 (66.1–84.2)	−16.8 pp (−27.4 to −3.6)	−37.3 to +6.2 pp
Burden D1	Any interval shortening, %	Week 52	27.3 (21.7–33.2)	11.5 (7.1–16.4)	+15.8 pp (+8.8 to +23.5)	Calibration-error-expanded sensitivity range includes zero
Burden D1	Any interval shortening, %	Week 104	48.2 (39.7–57.8)	27.1 (18.4–36.8)	+21.0 pp (+8.5 to +34.1)	Calibration-error-expanded sensitivity range includes zero

Efficacy rows are anchored relative contrasts and do not have modelled arm-level absolute means. Burden rows are conditional outputs from D1; protocol standardisation applies to these burden rows only. For efficacy, positive values denote greater BCVA gain or retinal-thickness reduction with faricimab; an interval spanning zero denotes no conclusive modelled difference. Positive injection and shortening values and negative interval values indicate lower modelled burden with aflibercept 8 mg. UI, uncertainty interval; pp, percentage points.

**FIGURE 2:**
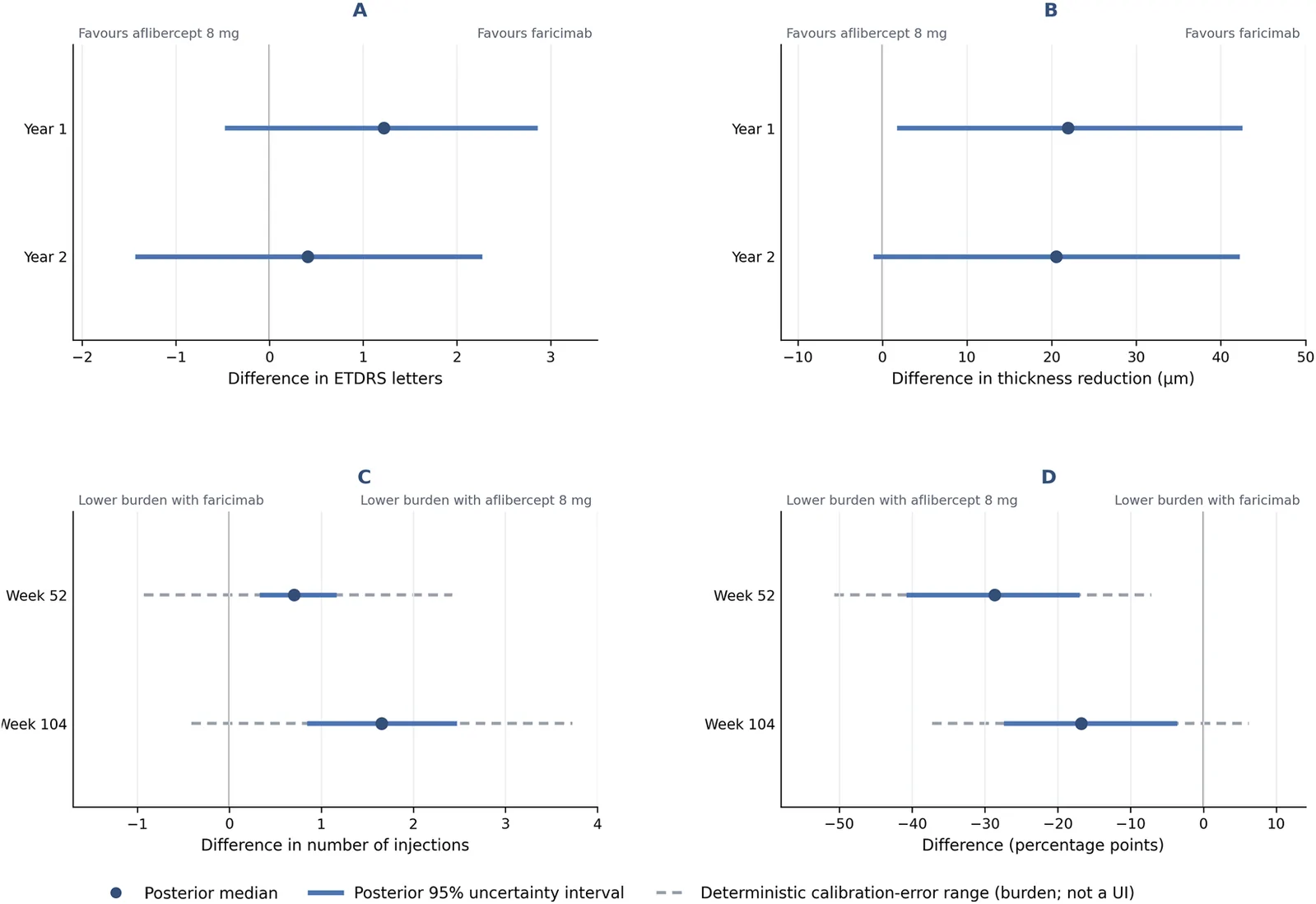
Modelled faricimab-minus-aflibercept 8 mg contrasts on separate scales. Points are posterior medians and blue lines are 95% uncertainty intervals. Grey dashed lines in burden panels are deterministic calibration-error-expanded ranges, not posterior uncertainty intervals. For efficacy, positive values denote greater BCVA gain or retinal-thickness reduction with faricimab; intervals spanning zero indicate no conclusive modelled difference. Positive injection values and negative Q16W values indicate lower modelled burden with aflibercept 8 mg. **(A) **Anchored BCVA comparison.** (B)** Anchored retinal-thickness comparison. **(C) **Conditional cumulative injections. **(D)** Conditional proportion at Q16W.

At year 1, the modelled difference in retinal-thickness reduction was +21.93 µm (95% UI, +1.70 to +42.52; P[Δ > 0] = 98.3%). At year 2, no conclusive modelled anatomical difference was identified (+20.53 µm; −1.08 to +42.24; P[Δ > 0] = 97.0%) because the interval included zero. Structural estimates ranged from +9.1 to +36.3 µm and +7.4 to +34.0 µm, respectively; magnitude was specification-dependent and clinical relevance uncertain (Table 3; Figure 2B; Supplementary Table S1).

### Conditional treatment burden

In the reference treatment-burden scenario (D1), mean cumulative injections at week 52 were 8.01 (95% UI, 7.70–8.43) with faricimab and 7.29 (7.13–7.58) with aflibercept 8 mg. By week 104, corresponding values were 12.60 (11.96–13.40) and 10.93 (10.60–11.48). Nominal differences were +0.71 injections at week 52 (+0.33 to +1.17) and +1.66 at week 104 (+0.84 to +2.47), where positive values indicate more injections with faricimab (Table 3; Figure 2C).

At week 52, modelled Q16W proportions were 50.9% with faricimab and 80.2% with aflibercept 8 mg; at week 104 they were 60.4% and 77.7%. Nominal differences were −28.7 percentage points (−40.8 to −17.0) and −16.8 percentage points (−27.4 to −3.6). Corresponding Q12W-or-longer proportions were 73.4% versus 90.7% and 79.0% versus 89.2%. Any interval shortening occurred in 27.3% versus 11.5% by week 52 and 48.2% versus 27.1% by week 104 (Table 3; Figure 2D).

Across ten non-null durability scenarios, the median injection difference ranged from +0.55 to +0.88 at week 52 and +1.32 to +2.04 at week 104. The combined nominal scenario envelope was +0.21 to +1.41 and +0.51 to +3.02 injections, respectively. All non-null scenarios nominally indicated fewer injections with aflibercept 8 mg (Supplementary Table S6).

After conservative expansion for original-regimen replication error, the injection-difference sensitivity ranges were −0.93 to +2.42 at week 52 and −0.41 to +3.73 at week 104. The week-104 Q16W sensitivity range also included zero. Thus, lower modelled burden with aflibercept 8 mg was a conditional scenario result and was not robust to conservative allowance for original-regimen replication error.

### Model qualification and external plausibility

All 13 critical validation targets were met, as were 15 of 17 targets overall. The two unmet exploratory targets concerned assigned 24-week intervals in PHOTON. The largest absolute injection replication errors were 0.57 for faricimab and 0.69 for aflibercept 8 mg. Supplementary Figure S1 presents 13 critical injection-count and dosing-interval validation targets (T1–T13) and four exploratory PHOTON extended-interval targets (T14–T17). Supplementary Table S4 provides the observed and model-predicted values, acceptance threshold, prediction error and whether each target was met.

Agreement with external plausibility targets was mixed, with one target within the specified tolerance and two outside it. At 12 months, predicted injection counts were 1.61 higher than FARWIDE-DMO for faricimab and 0.68 higher than the Swiss report for aflibercept 8 mg. The predicted proportion unable to maintain intervals of at least 7 weeks differed from Barbosa et al. by 30.4 percentage points (Barbosa et al., 2026; Peto et al., 2025; Spindler et al., 2026; Table 4). The burden estimates are therefore best interpreted within a fully adherent, trial-like setting and should not be directly extrapolated to routine practice or service capacity.

**TABLE 4:** External plausibility assessment.

Study	Treatment	Outcome	Observed	Predicted	Absolute error	Tolerance	Result
FARWIDE-DMO	Faricimab	Mean injections by 12 months	6.40	8.01 (7.70–8.43)	1.61 injections	1.50	Borderline outside tolerance
Swiss Retina Research Network	Aflibercept 8 mg	Mean injections by 12 months	6.60	7.28 (7.13–7.57)	0.68 injections	1.50	Within tolerance
Barbosa et al.	Aflibercept 8 mg	Unable to maintain ≥7-week interval by week 24	32.0%	1.6% (0.1%–5.7%)	30.4 pp	15 pp	Outside tolerance

External data were not used to estimate or recalibrate treatment contrasts. Failure of two targets limits generalisation to routine practice. pp, percentage points.

### Descriptive safety

Across faricimab source arms through week 100 (n = 1,262), reported ocular events included intraocular inflammation in 20 eyes (1.6%), endophthalmitis in six (0.5%) and no retinal vasculitis; 64 patients (5.1%) had an Antiplatelet Trialists’ Collaboration event and 31 (2.5%) died (Wong et al., 2024). Across aflibercept 8 mg source arms through week 96 (n = 491), intraocular inflammation occurred in six eyes (1.2%), with no reported endophthalmitis or occlusive retinal vasculitis; 33 patients (6.7%) had an Antiplatelet Trialists’ Collaboration event and 23 (4.7%) died (Do et al., 2026). Intraocular-pressure findings were summarised descriptively; no cross-programme inference was made (Table 5; Supplementary Table S8).

**TABLE 5:** Descriptive, non-comparative safety summary from the source programmes.

Source group	Window	N	IOI	Retinal vasculitis	Endophthalmitis	APTC event	Death	IOP/discontinuation
Faricimab 6 mg source arms	YOSEMITE/RHINE through week 100	1,262	20 (1.6%)	0	6 (0.5%)	64 (5.1%)	31 (2.5%)	No sustained IOP signal reported
Aflibercept 8 mg source arms	PHOTON through week 96	491	6 (1.2%)	0 reported	0 reported	33 (6.7%)	23 (4.7%)	No sustained IOP signal reported
All YOSEMITE/RHINE arms	Through year 1	1,891	—	—	—	—	—	141/1,891 (7.5%) discontinued treatment; 27/1,887 (1.4%) due to AE
All PHOTON arms	Through week 48	660	—	—	—	—	—	47/660 (7.1%) discontinued treatment; 5/658 (0.8%) due to AE

This table is descriptive only. Cross-programme differences in eligibility, follow-up, exposure and ascertainment preclude comparative safety inference. AE, adverse event; APTC, Antiplatelet Trialists’ Collaboration; IOI, intraocular inflammation; IOP, intraocular pressure.

## Discussion

This analysis addresses two distinct questions. The anchored comparison found no conclusive modelled BCVA difference. The year-1 anatomical reference interval excluded zero, whereas the year-2 interval included zero; both estimates varied across structures and had uncertain clinical relevance. Under the common virtual treat-and-extend protocol, aflibercept 8 mg was estimated to require fewer injections and achieve longer dosing intervals across all non-null scenarios. However, these estimates depended on durability data from trials with different source protocols, and the adjusted ranges included the possibility of no difference between treatments.

This separation is essential. BCVA and retinal-thickness estimates were assigned from the indirect comparison; they did not arise from simulated injections, adherence, shortening or disease-activity history. The analysis therefore does not estimate efficacy under a common treat-and-extend regimen. Conversely, the burden component estimates what the scheduling algorithm would produce if the transported latent durability distributions were applicable under identical decision rules. It cannot identify an intrinsic drug-specific durability effect. Protocol standardisation therefore applies to the burden component alone: it applies the same simulated scheduling rules to both treatments but does not create a common-protocol efficacy estimand.

This differs from the Bayesian network meta-analysis by Friedman et al., which estimated efficacy and injection burden under source-trial regimens (Friedman et al., 2025; Friedman et al., 2026). The present study retains regimen-level efficacy evidence but separately examines projected burden after loading, interval ceilings and adjustment rules are held constant. These efficacy findings are not independent validation because the analyses rely largely on the same pivotal trials; the main additional contribution is the direct evaluation of how dosing-protocol assumptions influence the comparison.

Two small retrospective direct cohorts provide useful short-term context but not a common-protocol or long-term burden estimate. Maccauro et al. included 28 eyes from 18 patients under retrospective PRN care with unequal loading, and Menna et al. included 60 treatment-naïve eyes with treatment selected by the physician and drug availability; neither found a statistically conclusive BCVA or retinal-thickness difference (Maccauro et al., 2025; Menna et al., 2026).

The BCVA findings are compatible with the pivotal trials, in which both newer regimens preserved visual benefit relative to aflibercept 2 mg (Brown et al., 2024; Do et al., 2026; Wong et al., 2024; Wykoff et al., 2022), and with the network meta-analysis (Friedman et al., 2025; Friedman et al., 2026). An interval containing zero does not establish equivalence. Although most posterior mass lay within ±3 letters, that range was not a prospectively justified equivalence margin. The estimated BCVA difference was small, with the uncertainty interval including no difference between treatments.

The year-1 anatomical reference estimate was positive, but the year-2 interval included zero. This pattern is directionally consistent with several YOSEMITE/RHINE analyses and the overlapping week-12 matching-adjusted comparison (Lim et al., 2025; Lim et al., 2026; Wong et al., 2024; Wykoff et al., 2022). Yet CST and CRT differed in segmentation boundary and device reporting, and the 7–36 µm structural range was wide. Retinal thickness is an incomplete surrogate for visual function (Diabetic Retinopathy Clinical Research Network, 2007; Nanegrungsunk et al., 2022; Wang et al., 2023). These estimates do not establish clinical superiority.

The burden analysis is more vulnerable to cross-protocol transport. Faricimab intervals were progressively achieved after response-dependent personalised decisions, whereas aflibercept 8 mg intervals were assigned at Q12W or Q16W and maintained or shortened. Discounting the likelihood widens posterior uncertainty but cannot remove a directional protocol bias. The D1–D10 envelope exposes this dependence, and conservative expansion for original-regimen replication error further shows that prediction error is large relative to the week-52 contrast. These results may motivate a head-to-head trial but should not guide a claim that one molecule inherently requires fewer injections.

The cross-trial table also identifies a fundamental limitation of the anchored comparison. The common aflibercept 2 mg groups were similar on several averages, but prior anti-VEGF exposure differed materially, HbA1c and reported disease-duration measures were not identical, OCT definitions differed and some variables were unreported. Observed aflibercept 2 mg responses also differed across programmes. Anchoring preserves within-trial randomisation for the observed contrasts but does not repair unmeasured effect modification between trials. The assumption that the trials were sufficiently comparable for indirect comparison could not be fully confirmed.

The external comparisons were plausibility checks of applicability, not validation of the relative treatment effect. Clinician decisions about monitoring, retreatment, extension, shortening and delayed treatment are among the reasons why routine-practice patterns can differ from a fully adherent trial-like schedule. The observed failures therefore restrict the simulator to the latter setting.

Safety was not simulated and cannot be inferred from efficacy or scheduling. The descriptive source summaries do not permit a cross-trial safety ranking because exposure, follow-up, eligibility and ascertainment differ. Treatment choice must still consider labelled ocular and systemic risks, patient characteristics and direct safety evidence.

The principal methodological contribution is clear separation of anchored efficacy from a common-algorithm burden simulation, which examines projected injections and achieved intervals after loading, interval limits and adjustment rules are held constant. Strengths include within-trial anchored contrasts, covariance for shared comparators, full numerical source tables, labelled qualification assessment, alternative priors and efficacy structures, one-, two- and three-component durability structures, composite leave-one-trial/regimen-out stress tests, replication-error propagation, reproducible code and exact reproduction checks. The full and quick runs showed the same substantive pattern, indicating that Monte Carlo noise did not drive the findings.

Several limitations should be considered. The use of aggregate data prevented adjustment for patient-level effect modifiers and stratification by previous treatment. The weak week-12 prior was derived from a published, manufacturer-supported matching-adjusted indirect comparison involving participants from the same pivotal trial programmes; no matching was performed in the present study, and only measured baseline differences could be addressed. Retinal-thickness outcomes could not be made fully comparable because the source studies used different OCT measures and reporting methods. The durability model represented the ability to extend dosing intervals rather than longitudinal changes in BCVA, retinal thickness, fluid or DRSS. The validation tolerances assessed reproduction of source-trial outcomes and should not be interpreted as measures of precision in the between-treatment comparison. Although all 13 critical validation targets were met, the two exploratory Q24 targets and two of the three external plausibility targets were outside their specified tolerances.

A prospective head-to-head randomised trial remains necessary. It should stratify prior treatment exposure, use identical loading and modification rules, harmonise OCT acquisition and segmentation, and prespecify visual, anatomical, fluid, DRSS, injection, safety and patient-reported burden outcomes. Until then, the present estimates should be interpreted as exploratory.

## Conclusion

The anchored aggregate-data comparison showed small estimated BCVA differences between faricimab 6 mg and aflibercept 8 mg, with the uncertainty intervals including no treatment difference. In the reference analysis, faricimab showed a greater estimated reduction in retinal thickness at year 1, whereas the year-2 estimate was compatible with no difference. The magnitude of the anatomical difference varied across model specifications, and its clinical relevance remains uncertain. Under the common virtual treat-and-extend protocol, the separate microsimulation estimated fewer injections with aflibercept 8 mg across scenarios in which the treatments were allowed to differ in durability. However, after accounting for calibration uncertainty, the adjusted ranges included the possibility of no difference between treatments.

## Data Availability

The original contributions presented in the study are included in the article/Supplementary Material, further inquiries can be directed to the corresponding author.

## Supplementary material

SUPPLEMENTARY FIGURE S1 Original-regimen qualification targets. Absolute observed–predicted error is divided by the engineering tolerance specified and locked after peer review but before the final reviewer-driven reanalysis. T1–T17 are classified as critical or exploratory; Supplementary Table S4 provides observed and predicted values, errors, tolerances and pass status.

SUPPLEMENTARY FIGURE S2 Structural sensitivity of the anchored efficacy comparison. Panels separate BCVA and anatomy at years 1 and 2; points are posterior medians and lines are 95% uncertainty intervals. E7–E10 are composite stress tests that omit the nominal-interval covariate, the week-12 prior and the named trial or regimen. All BCVA intervals include zero; anatomical magnitude is specification-dependent.
