# Supplementary material for: Faricimab 6 mg versus aflibercept 8 mg in diabetic macular oedema: an in silico study of anchored efficacy and common-protocol treatment burden
Source: Front Pharmacol. 2026 Oct 13;17:1938908. doi: 10.3389/fphar.2026.1938908 (PMC13710820; doi:10.3389/fphar.2026.1938908)
Supplement: Supplementary file 1 [file Supplementaryfile1.zip › Supplementary_Material (revised v2.0).docx]

**Supplementary material**

This supplement contains complete numerical inputs, qualification results, structural sensitivity analyses, cross-trial comparability, descriptive safety extraction, computational details and evidence-search methods. Comparative values are faricimab minus aflibercept 8 mg unless otherwise stated.

**Supplementary Table S1. Structural sensitivity of the anchored efficacy comparison**

| **Analysis** | **Endpoint** | **Year** | **Median** | **95% UI** | **P(Δ>0)** | **P(\|BCVA Δ\|<3)** | **P(Δ > endpoint-specific threshold)†** | **Draws** |
| --- | --- | --- | --- | --- | --- | --- | --- | --- |
| **E0 reference structure** | Anatomy | 1 | +22.46 | +2.44 to +43.02 | 98.3% | — | 60.0% | 3000 |
| **E0 reference structure** | Anatomy | 2 | +19.93 | -2.13 to +41.75 | 96.4% | — | 49.7% | 3000 |
| **E0 reference structure** | BCVA | 1 | +1.19 | -0.49 to +2.88 | 92.2% | 98.3% | 1.7% | 3000 |
| **E0 reference structure** | BCVA | 2 | +0.44 | -1.32 to +2.28 | 69.7% | 99.6% | 0.4% | 3000 |
| **E1 no week-12 prior** | Anatomy | 1 | +22.59 | -1.58 to +48.95 | 96.7% | — | 58.7% | 3000 |
| **E1 no week-12 prior** | Anatomy | 2 | +19.15 | -6.17 to +45.40 | 93.2% | — | 47.5% | 3000 |
| **E1 no week-12 prior** | BCVA | 1 | +1.33 | -0.68 to +3.38 | 90.2% | 94.9% | 5.1% | 3000 |
| **E1 no week-12 prior** | BCVA | 2 | +0.22 | -2.07 to +2.40 | 58.4% | 99.0% | 0.7% | 3000 |
| **E2 sceptical zero prior** | Anatomy | 1 | +9.11 | -6.74 to +24.48 | 87.0% | — | 8.3% | 3000 |
| **E2 sceptical zero prior** | Anatomy | 2 | +7.36 | -8.17 to +23.39 | 82.7% | — | 5.8% | 3000 |
| **E2 sceptical zero prior** | BCVA | 1 | +0.68 | -0.73 to +2.02 | 83.5% | 99.9% | 0.1% | 3000 |
| **E2 sceptical zero prior** | BCVA | 2 | +0.06 | -1.41 to +1.58 | 53.5% | 100.0% | 0.0% | 3000 |
| **E3 no interval covariate** | Anatomy | 1 | +25.00 | +5.00 to +44.49 | 99.3% | — | 69.8% | 3000 |
| **E3 no interval covariate** | Anatomy | 2 | +23.48 | +1.02 to +44.96 | 98.2% | — | 62.1% | 3000 |
| **E3 no interval covariate** | BCVA | 1 | +1.29 | -0.28 to +2.90 | 93.9% | 98.3% | 1.7% | 3000 |
| **E3 no interval covariate** | BCVA | 2 | +0.56 | -1.19 to +2.25 | 73.2% | 99.5% | 0.5% | 3000 |
| **E4 no interval covariate/no prior** | Anatomy | 1 | +27.20 | +1.44 to +52.03 | 98.1% | — | 71.5% | 3000 |
| **E4 no interval covariate/no prior** | Anatomy | 2 | +23.55 | -2.09 to +50.61 | 96.5% | — | 60.4% | 3000 |
| **E4 no interval covariate/no prior** | BCVA | 1 | +1.47 | -0.45 to +3.47 | 93.1% | 94.4% | 5.6% | 3000 |
| **E4 no interval covariate/no prior** | BCVA | 2 | +0.36 | -1.75 to +2.48 | 62.5% | 98.7% | 1.1% | 3000 |
| **E5 treatment-specific slopes** | Anatomy | 1 | +22.64 | +2.96 to +42.81 | 99.1% | — | 60.8% | 3000 |
| **E5 treatment-specific slopes** | Anatomy | 2 | +20.11 | -0.82 to +42.61 | 96.9% | — | 50.3% | 3000 |
| **E5 treatment-specific slopes** | BCVA | 1 | +1.21 | -0.48 to +2.86 | 92.9% | 98.3% | 1.7% | 3000 |
| **E5 treatment-specific slopes** | BCVA | 2 | +0.49 | -1.32 to +2.29 | 71.0% | 99.6% | 0.4% | 3000 |
| **E6 fixed effect** | Anatomy | 1 | +22.36 | +3.78 to +40.71 | 99.0% | — | 60.6% | 3000 |
| **E6 fixed effect** | Anatomy | 2 | +19.57 | -1.09 to +40.34 | 97.0% | — | 48.1% | 3000 |
| **E6 fixed effect** | BCVA | 1 | +1.22 | -0.31 to +2.72 | 93.7% | 98.9% | 1.1% | 3000 |
| **E6 fixed effect** | BCVA | 2 | +0.38 | -1.42 to +2.13 | 66.8% | 99.7% | 0.3% | 3000 |
| E7 no interval/no prior; omit YOSEMITE | Anatomy | 1 | +22.99 | -5.80 to +50.87 | 94.0% | — | 58.0% | 3000 |
| E7 no interval/no prior; omit YOSEMITE | Anatomy | 2 | +23.25 | -6.02 to +52.78 | 94.3% | — | 59.1% | 3000 |
| E7 no interval/no prior; omit YOSEMITE | BCVA | 1 | +1.74 | -0.66 to +3.94 | 92.9% | 87.1% | 12.8% | 3000 |
| E7 no interval/no prior; omit YOSEMITE | BCVA | 2 | +1.20 | -1.42 to +3.80 | 83.5% | 91.5% | 8.5% | 3000 |
| E8 no interval/no prior; omit RHINE | Anatomy | 1 | +31.47 | +1.38 to +59.74 | 97.9% | — | 78.4% | 3000 |
| E8 no interval/no prior; omit RHINE | Anatomy | 2 | +23.14 | -8.68 to +54.03 | 92.8% | — | 57.4% | 3000 |
| E8 no interval/no prior; omit RHINE | BCVA | 1 | +1.06 | -1.31 to +3.41 | 82.0% | 95.1% | 4.8% | 3000 |
| E8 no interval/no prior; omit RHINE | BCVA | 2 | -0.46 | -3.07 to +2.12 | 36.7% | 96.9% | 0.4% | 3000 |
| E9 no interval/no prior; omit PHOTON Q12W | Anatomy | 1 | +36.30 | +6.33 to +64.64 | 98.9% | — | 85.3% | 3000 |
| E9 no interval/no prior; omit PHOTON Q12W | Anatomy | 2 | +34.02 | +3.79 to +63.45 | 98.7% | — | 82.3% | 3000 |
| E9 no interval/no prior; omit PHOTON Q12W | BCVA | 1 | +1.76 | -0.54 to +3.91 | 93.2% | 86.8% | 13.1% | 3000 |
| E9 no interval/no prior; omit PHOTON Q12W | BCVA | 2 | +0.89 | -1.58 to +3.30 | 77.0% | 95.8% | 3.9% | 3000 |
| E10 no interval/no prior; omit PHOTON Q16W | Anatomy | 1 | +19.65 | -8.17 to +45.42 | 92.0% | — | 48.9% | 3000 |
| E10 no interval/no prior; omit PHOTON Q16W | Anatomy | 2 | +14.50 | -12.16 to +40.83 | 85.5% | — | 33.6% | 3000 |
| E10 no interval/no prior; omit PHOTON Q16W | BCVA | 1 | +1.07 | -1.14 to +3.20 | 83.6% | 96.4% | 3.6% | 3000 |
| E10 no interval/no prior; omit PHOTON Q16W | BCVA | 2 | -0.11 | -2.58 to +2.19 | 46.5% | 98.5% | 0.4% | 3000 |

†For the final probability column, the endpoint-specific threshold was +3 ETDRS letters for BCVA and +20 µm for anatomy. P(|BCVA Δ|<3) is descriptive and does not establish equivalence. Positive anatomical contrasts indicate a greater reduction in retinal thickness with faricimab. All BCVA 95% uncertainty intervals included zero; anatomical intervals that included zero were compatible with no between-treatment difference.

**Supplementary Table S2. Complete anchored-efficacy inputs**

| **Contrast** | **Endpoint** | **Year** | **Trial** | **Regimen** | **Interval** | **Estimate** | **SE** | **SE source** | **Comparator cluster** | **Transformation** | **SE derivation** |
| --- | --- | --- | --- | --- | --- | --- | --- | --- | --- | --- | --- |
| far_q8_vs_afl2 | bcva | 1 | YOSEMITE | faricimab q8w | 8 | -0.2 | 0.8020 | reported | YOSEMITE | Within-trial active-minus-aflibercept-2-mg contrast. | Derived from the reported confidence interval or directly reported uncertainty. |
| far_q8_vs_afl2 | bcva | 1 | RHINE | faricimab q8w | 8 | 1.5 | 0.7351 | reported | RHINE | Within-trial active-minus-aflibercept-2-mg contrast. | Derived from the reported confidence interval or directly reported uncertainty. |
| far_pti_vs_afl2 | bcva | 1 | YOSEMITE | faricimab PTI | 13.3 | 0.7 | 0.8020 | reported | YOSEMITE | Within-trial active-minus-aflibercept-2-mg contrast. | Derived from the reported confidence interval or directly reported uncertainty. |
| far_pti_vs_afl2 | bcva | 1 | RHINE | faricimab PTI | 13.3 | 0.5 | 0.7129 | reported | RHINE | Within-trial active-minus-aflibercept-2-mg contrast. | Derived from the reported confidence interval or directly reported uncertainty. |
| afl8_q12_vs_afl2 | bcva | 1 | PHOTON | aflibercept 8 mg q12w | 12 | -0.57 | 0.8648 | reported | PHOTON | Within-trial active-minus-aflibercept-2-mg contrast. | Derived from the reported confidence interval or directly reported uncertainty. |
| afl8_q16_vs_afl2 | bcva | 1 | PHOTON | aflibercept 8 mg q16w | 16 | -1.44 | 0.9337 | reported | PHOTON | Within-trial active-minus-aflibercept-2-mg contrast. | Derived from the reported confidence interval or directly reported uncertainty. |
| far_q8_vs_afl2 | bcva | 2 | YOSEMITE | faricimab q8w | 8 | -0.7 | 1.0000 | approximated | YOSEMITE | Within-trial active-minus-aflibercept-2-mg contrast. | Approximate standard error used because a directly reported contrast SE was unavailable; tested with 1.5-fold inflation. |
| far_q8_vs_afl2 | bcva | 2 | RHINE | faricimab q8w | 8 | 1.5 | 1.0000 | approximated | RHINE | Within-trial active-minus-aflibercept-2-mg contrast. | Approximate standard error used because a directly reported contrast SE was unavailable; tested with 1.5-fold inflation. |
| far_pti_vs_afl2 | bcva | 2 | YOSEMITE | faricimab PTI | 13.3 | -0.7 | 1.0000 | approximated | YOSEMITE | Within-trial active-minus-aflibercept-2-mg contrast. | Approximate standard error used because a directly reported contrast SE was unavailable; tested with 1.5-fold inflation. |
| far_pti_vs_afl2 | bcva | 2 | RHINE | faricimab PTI | 13.3 | 0.7 | 1.0000 | approximated | RHINE | Within-trial active-minus-aflibercept-2-mg contrast. | Approximate standard error used because a directly reported contrast SE was unavailable; tested with 1.5-fold inflation. |
| afl8_q12_vs_afl2 | bcva | 2 | PHOTON | aflibercept 8 mg q12w | 12 | 0.4 | 1.0000 | approximated | PHOTON | Within-trial active-minus-aflibercept-2-mg contrast. | Approximate standard error used because a directly reported contrast SE was unavailable; tested with 1.5-fold inflation. |
| afl8_q16_vs_afl2 | bcva | 2 | PHOTON | aflibercept 8 mg q16w | 16 | -0.9 | 1.0000 | approximated | PHOTON | Within-trial active-minus-aflibercept-2-mg contrast. | Approximate standard error used because a directly reported contrast SE was unavailable; tested with 1.5-fold inflation. |
| far_q8_vs_afl2 | anatomy | 1 | YOSEMITE | faricimab q8w | 8 | 36.3 | 5.8704 | reported | YOSEMITE | Within-trial active-minus-aflibercept-2-mg contrast; sign harmonised so positive denotes greater thickness reduction with the active regimen. | Derived from the reported confidence interval or directly reported uncertainty. |
| far_q8_vs_afl2 | anatomy | 1 | RHINE | faricimab q8w | 8 | 25.7 | 5.9604 | reported | RHINE | Within-trial active-minus-aflibercept-2-mg contrast; sign harmonised so positive denotes greater thickness reduction with the active regimen. | Derived from the reported confidence interval or directly reported uncertainty. |
| far_pti_vs_afl2 | anatomy | 1 | YOSEMITE | faricimab PTI | 13.3 | 26.2 | 5.8704 | reported | YOSEMITE | Within-trial active-minus-aflibercept-2-mg contrast; sign harmonised so positive denotes greater thickness reduction with the active regimen. | Derived from the reported confidence interval or directly reported uncertainty. |
| far_pti_vs_afl2 | anatomy | 1 | RHINE | faricimab PTI | 13.3 | 17.5 | 5.9065 | reported | RHINE | Within-trial active-minus-aflibercept-2-mg contrast; sign harmonised so positive denotes greater thickness reduction with the active regimen. | Derived from the reported confidence interval or directly reported uncertainty. |
| afl8_q12_vs_afl2 | anatomy | 1 | PHOTON | aflibercept 8 mg q12w | 12 | 6.4 | 13.3695 | reported | PHOTON | Within-trial active-minus-aflibercept-2-mg contrast; sign harmonised so positive denotes greater thickness reduction with the active regimen. | Derived from the reported confidence interval or directly reported uncertainty. |
| afl8_q16_vs_afl2 | anatomy | 1 | PHOTON | aflibercept 8 mg q16w | 16 | -17 | 15.0515 | reported | PHOTON | Within-trial active-minus-aflibercept-2-mg contrast; sign harmonised so positive denotes greater thickness reduction with the active regimen. | Derived from the reported confidence interval or directly reported uncertainty. |
| far_q8_vs_afl2 | anatomy | 2 | YOSEMITE | faricimab q8w | 8 | 19.7 | 5.7803 | reported | YOSEMITE | Within-trial active-minus-aflibercept-2-mg contrast; sign harmonised so positive denotes greater thickness reduction with the active regimen. | Derived from the reported confidence interval or directly reported uncertainty. |
| far_q8_vs_afl2 | anatomy | 2 | RHINE | faricimab q8w | 8 | 17 | 6.1045 | reported | RHINE | Within-trial active-minus-aflibercept-2-mg contrast; sign harmonised so positive denotes greater thickness reduction with the active regimen. | Derived from the reported confidence interval or directly reported uncertainty. |
| far_pti_vs_afl2 | anatomy | 2 | YOSEMITE | faricimab PTI | 13.3 | 8.2 | 5.7624 | reported | YOSEMITE | Within-trial active-minus-aflibercept-2-mg contrast; sign harmonised so positive denotes greater thickness reduction with the active regimen. | Derived from the reported confidence interval or directly reported uncertainty. |
| far_pti_vs_afl2 | anatomy | 2 | RHINE | faricimab PTI | 13.3 | 11.5 | 6.0154 | reported | RHINE | Within-trial active-minus-aflibercept-2-mg contrast; sign harmonised so positive denotes greater thickness reduction with the active regimen. | Derived from the reported confidence interval or directly reported uncertainty. |
| afl8_q12_vs_afl2 | anatomy | 2 | PHOTON | aflibercept 8 mg q12w | 12 | -1.7 | 13.9140 | reported | PHOTON | Within-trial active-minus-aflibercept-2-mg contrast; sign harmonised so positive denotes greater thickness reduction with the active regimen. | Derived from the reported confidence interval or directly reported uncertainty. |
| afl8_q16_vs_afl2 | anatomy | 2 | PHOTON | aflibercept 8 mg q16w | 16 | -32 | 16.0305 | reported | PHOTON | Within-trial active-minus-aflibercept-2-mg contrast; sign harmonised so positive denotes greater thickness reduction with the active regimen. | Derived from the reported confidence interval or directly reported uncertainty. |

*Every efficacy contrast, standard error and transformation used in the analysis is shown. SE, standard error.*

**Supplementary Table S3. Complete durability evidence**

| **Drug** | **Model week** | **Source week** | **Likelihood** | **Category** | **Count** | **Success** | **Total** | **Threshold** | **Weight** | **Source** | **Transformation** |
| --- | --- | --- | --- | --- | --- | --- | --- | --- | --- | --- | --- |
| faricimab | 52 | 52 | category_count | q4 | 72 | — | — | — | 1 | Pooled YOSEMITE/RHINE PTI | Pooled category count used in a power-discounted multinomial likelihood. |
| faricimab | 52 | 52 | category_count | q8 | 92 | — | — | — | 1 | Pooled YOSEMITE/RHINE PTI | Pooled category count used in a power-discounted multinomial likelihood. |
| faricimab | 52 | 52 | category_count | q12 | 122 | — | — | — | 1 | Pooled YOSEMITE/RHINE PTI | Pooled category count used in a power-discounted multinomial likelihood. |
| faricimab | 52 | 52 | category_count | q16 | 308 | — | — | — | 1 | Pooled YOSEMITE/RHINE PTI | Pooled category count used in a power-discounted multinomial likelihood. |
| faricimab | 96 | 96 | category_count | lt12 | 122 | — | — | — | 1 | Pooled YOSEMITE/RHINE PTI; q4/q8 not separately reported | Pooled category count used in a power-discounted multinomial likelihood. |
| faricimab | 96 | 96 | category_count | q12 | 88 | — | — | — | 1 | Pooled YOSEMITE/RHINE PTI | Pooled category count used in a power-discounted multinomial likelihood. |
| faricimab | 96 | 96 | category_count | q16 | 347 | — | — | — | 1 | Pooled YOSEMITE/RHINE PTI | Pooled category count used in a power-discounted multinomial likelihood. |
| aflibercept8 | 52 | 48 | binomial_tail | ge12 | — | 273 | 300 | 10 | 1 | PHOTON q12 arm maintained q12 through week 48 | Success/total used in a power-discounted binomial tail likelihood. |
| aflibercept8 | 52 | 48 | binomial_tail | ge16 | — | 139 | 156 | 14 | 1 | PHOTON q16 arm maintained q16 through week 48 | Success/total used in a power-discounted binomial tail likelihood. |
| aflibercept8 | 96 | 96 | binomial_tail | ge12 | — | 225 | 256 | 10 | 1 | PHOTON q12 arm maintained at least q12 through week 96; count reconstructed from 88% | Success/total used in a power-discounted binomial tail likelihood. |
| aflibercept8 | 96 | 96 | binomial_tail | ge16 | — | 115 | 139 | 14 | 1 | PHOTON q16 arm maintained at least q16 through week 96; count reconstructed from 83% | Success/total used in a power-discounted binomial tail likelihood. |
| aflibercept8 | 96 | 96 | binomial_tail | ge20 | — | 110 | 256 | 18 | 0.35 | PHOTON q12 arm last assigned >=Q20; assigned/qualified rather than necessarily completed | Success/total used in a power-discounted binomial tail likelihood. |
| aflibercept8 | 96 | 96 | binomial_tail | ge20 | — | 65 | 139 | 18 | 0.35 | PHOTON q16 arm last assigned >=Q20; assigned/qualified rather than necessarily completed | Success/total used in a power-discounted binomial tail likelihood. |
| aflibercept8 | 96 | 96 | binomial_tail | q24 | — | 61 | 256 | 22 | 0.35 | PHOTON q12 arm last assigned Q24; assigned/qualified rather than necessarily completed | Success/total used in a power-discounted binomial tail likelihood. |
| aflibercept8 | 96 | 96 | binomial_tail | q24 | — | 45 | 139 | 22 | 0.35 | PHOTON q16 arm last assigned Q24; assigned/qualified rather than necessarily completed | Success/total used in a power-discounted binomial tail likelihood. |

*Assigned, maintained and achieved interval evidence is not assumed to represent the same latent construct; alternative readings are evaluated in D1–D10.*

**Supplementary Table S4. Complete model-qualification assessment**

| **No.** | **Target** | **Drug** | **Week** | **Metric** | **Observed** | **Predicted median** | **Predicted 95% interval** | **Absolute error** | **Tolerance** | **Designation** | **Result** | **Source** | **Interpretation** |
| --- | --- | --- | --- | --- | --- | --- | --- | --- | --- | --- | --- | --- | --- |
| 1 | far_pti_injections_100 | faricimab | 100 | mean_injections | 11.80 | 12.37 | 11.75 to 13.10 | 0.57 | 1.00 | Critical | Pass | Pooled YOSEMITE/RHINE means | Injection-burden replication under the original trial regimen. |
| 2 | far_pti_ge12_52 | faricimab | 52 | p_ge_q12 | 72.46% | 73.10% | 65.23 to 80.83% | 0.64% | 10.00% | Critical | Pass | Pooled YOSEMITE/RHINE | Interval/durability replication under the original trial regimen. |
| 3 | far_pti_ge16_52 | faricimab | 52 | p_ge_q16 | 51.85% | 50.93% | 41.55 to 59.99% | 0.92% | 10.00% | Critical | Pass | Pooled YOSEMITE/RHINE | Interval/durability replication under the original trial regimen. |
| 4 | far_pti_ge12_96 | faricimab | 96 | p_ge_q12 | 78.00% | 78.43% | 72.08 to 84.93% | 0.43% | 10.00% | Critical | Pass | Pooled YOSEMITE/RHINE | Interval/durability replication under the original trial regimen. |
| 5 | far_pti_ge16_96 | faricimab | 96 | p_ge_q16 | 62.30% | 59.18% | 51.73 to 67.30% | 3.12% | 10.00% | Critical | Pass | Pooled YOSEMITE/RHINE | Interval/durability replication under the original trial regimen. |
| 6 | afl8_q12_injections_48 | aflibercept8 | 48 | mean_injections | 6.00 | 6.12 | 6.06 to 6.19 | 0.12 | 1.00 | Critical | Pass | PHOTON | Injection-burden replication under the original trial regimen. |
| 7 | afl8_q12_injections_96 | aflibercept8 | 96 | mean_injections | 9.50 | 9.72 | 9.56 to 9.90 | 0.22 | 1.00 | Critical | Pass | PHOTON | Injection-burden replication under the original trial regimen. |
| 8 | afl8_q12_maintained_48 | aflibercept8 | 48 | p_maintained | 91.00% | 91.03% | 85.85 to 95.08% | 0.03% | 10.00% | Critical | Pass | PHOTON | Interval/durability replication under the original trial regimen. |
| 9 | afl8_q12_maintained_96 | aflibercept8 | 96 | p_maintained | 88.00% | 85.17% | 79.17 to 90.17% | 2.83% | 10.00% | Critical | Pass | PHOTON | Interval/durability replication under the original trial regimen. |
| 10 | afl8_q16_injections_48 | aflibercept8 | 48 | mean_injections | 5.00 | 5.18 | 5.11 to 5.25 | 0.18 | 1.00 | Critical | Pass | PHOTON | Injection-burden replication under the original trial regimen. |
| 11 | afl8_q16_injections_96 | aflibercept8 | 96 | mean_injections | 7.80 | 8.49 | 8.29 to 8.67 | 0.69 | 1.00 | Critical | Pass | PHOTON | Injection-burden replication under the original trial regimen. |
| 12 | afl8_q16_maintained_48 | aflibercept8 | 48 | p_maintained | 89.00% | 84.67% | 78.27 to 91.35% | 4.33% | 10.00% | Critical | Pass | PHOTON | Interval/durability replication under the original trial regimen. |
| 13 | afl8_q16_maintained_96 | aflibercept8 | 96 | p_maintained | 83.00% | 76.27% | 68.50 to 83.63% | 6.73% | 10.00% | Critical | Pass | PHOTON | Interval/durability replication under the original trial regimen. |
| 14 | afl8_q12_ge20_96 | aflibercept8 | 96 | p_ge_q20 | 43.00% | 32.03% | 25.62 to 38.73% | 10.97% | 12.00% | Exploratory | Pass | PHOTON last assigned/qualified interval; not necessarily completed | Interval/durability replication under the original trial regimen. |
| 15 | afl8_q16_ge20_96 | aflibercept8 | 96 | p_ge_q20 | 47.00% | 49.50% | 38.73 to 60.40% | 2.50% | 12.00% | Exploratory | Pass | PHOTON last assigned/qualified interval; not necessarily completed | Interval/durability replication under the original trial regimen. |
| 16 | afl8_q12_q24_96 | aflibercept8 | 96 | p_q24 | 24.00% | 2.23% | 1.03 to 3.77% | 21.77% | 12.00% | Exploratory | Fail | PHOTON last assigned/qualified interval; not necessarily completed | Interval/durability replication under the original trial regimen. |
| 17 | afl8_q16_q24_96 | aflibercept8 | 96 | p_q24 | 32.00% | 9.03% | 4.40 to 14.80% | 22.97% | 12.00% | Exploratory | Fail | PHOTON last assigned/qualified interval; not necessarily completed | Interval/durability replication under the original trial regimen. |

All 13 critical qualification targets met their specified tolerances, as did 15 of 17 targets overall. The two targets outside tolerance were exploratory PHOTON Q24 assigned-interval targets. These tolerances assess model qualification and are not measures of inferential precision (Supplementary Table S4; Supplementary Figure S1).

**Supplementary Table S5. Cross-trial comparability evidence**

| **Trial** | **Arm** | **Timepoint** | **Variable** | **Value** | **Unit** | **Denominator** | **Statistic** | **Comment** |
| --- | --- | --- | --- | --- | --- | --- | --- | --- |
| PHOTON | aflibercept 2 mg q8w | baseline | age | 63.0 (9.8) | years | n=167 | mean (SD) | — |
| PHOTON | aflibercept 2 mg q8w | baseline | male | 92 (55%) | patients | n=167 | n (%) | — |
| PHOTON | aflibercept 2 mg q8w | baseline | HbA1c | 8.1 (1.5) | % | n=167 | mean (SD) | — |
| PHOTON | aflibercept 2 mg q8w | baseline | BCVA | 61.5 (11.2) | ETDRS letters | n=167 | mean (SD) | — |
| PHOTON | aflibercept 2 mg q8w | baseline | CRT | 457.2 (144.0) | µm | n=167 | mean (SD) | — |
| PHOTON | aflibercept 8 mg q12w | baseline | age | 62.1 (11.1) | years | n=328 | mean (SD) | — |
| PHOTON | aflibercept 8 mg q12w | baseline | male | 210 (64%) | patients | n=328 | n (%) | — |
| PHOTON | aflibercept 8 mg q12w | baseline | HbA1c | 7.9 (1.5) | % | n=328 | mean (SD) | — |
| PHOTON | aflibercept 8 mg q12w | baseline | BCVA | 63.6 (10.1) | ETDRS letters | n=328 | mean (SD) | — |
| PHOTON | aflibercept 8 mg q12w | baseline | CRT | 449.1 (127.4) | µm | n=328 | mean (SD) | — |
| PHOTON | aflibercept 8 mg q16w | baseline | age | 61.9 (9.5) | years | n=163 | mean (SD) | — |
| PHOTON | aflibercept 8 mg q16w | baseline | male | 99 (61%) | patients | n=163 | n (%) | — |
| PHOTON | aflibercept 8 mg q16w | baseline | HbA1c | 7.8 (1.5) | % | n=163 | mean (SD) | — |
| PHOTON | aflibercept 8 mg q16w | baseline | BCVA | 61.4 (11.8) | ETDRS letters | n=163 | mean (SD) | — |
| PHOTON | aflibercept 8 mg q16w | baseline | CRT | 460.3 (117.8) | µm | n=163 | mean (SD) | — |
| YOSEMITE | faricimab 6 mg q8w | baseline | age | 61.6 (9.5) | years | n=315 | mean (SD) | — |
| YOSEMITE | faricimab 6 mg q8w | baseline | male | 187 (59%) | patients | n=315 | n (%) | — |
| YOSEMITE | faricimab 6 mg q8w | baseline | HbA1c | 7.6 (1.1) | % | n=315 | mean (SD) | — |
| YOSEMITE | faricimab 6 mg q8w | baseline | BCVA | 62.0 (9.9) | ETDRS letters | n=315 | mean (SD) | — |
| YOSEMITE | faricimab 6 mg q8w | baseline | CST | 492.3 (135.8) | µm | n=315 | mean (SD) | — |
| YOSEMITE | faricimab 6 mg PTI/T&E | baseline | age | 62.8 (10.0) | years | n=313 | mean (SD) | — |
| YOSEMITE | faricimab 6 mg PTI/T&E | baseline | male | 197 (63%) | patients | n=313 | n (%) | — |
| YOSEMITE | faricimab 6 mg PTI/T&E | baseline | HbA1c | 7.6 (1.1) | % | n=313 | mean (SD) | — |
| YOSEMITE | faricimab 6 mg PTI/T&E | baseline | BCVA | 61.9 (10.2) | ETDRS letters | n=313 | mean (SD) | — |
| YOSEMITE | faricimab 6 mg PTI/T&E | baseline | CST | 485.8 (130.8) | µm | n=313 | mean (SD) | — |
| YOSEMITE | aflibercept 2 mg q8w | baseline | age | 62.2 (9.6) | years | n=312 | mean (SD) | — |
| YOSEMITE | aflibercept 2 mg q8w | baseline | male | 178 (57%) | patients | n=312 | n (%) | — |
| YOSEMITE | aflibercept 2 mg q8w | baseline | HbA1c | 7.6 (1.1) | % | n=312 | mean (SD) | — |
| YOSEMITE | aflibercept 2 mg q8w | baseline | BCVA | 62.2 (9.5) | ETDRS letters | n=312 | mean (SD) | — |
| YOSEMITE | aflibercept 2 mg q8w | baseline | CST | 484.5 (131.1) | µm | n=312 | mean (SD) | — |
| RHINE | faricimab 6 mg q8w | baseline | age | 62.5 (10.1) | years | n=317 | mean (SD) | — |
| RHINE | faricimab 6 mg q8w | baseline | male | 194 (61%) | patients | n=317 | n (%) | — |
| RHINE | faricimab 6 mg q8w | baseline | HbA1c | 7.6 (1.2) | % | n=317 | mean (SD) | — |
| RHINE | faricimab 6 mg q8w | baseline | BCVA | 61.9 (10.1) | ETDRS letters | n=317 | mean (SD) | — |
| RHINE | faricimab 6 mg q8w | baseline | CST | 466.2 (119.4) | µm | n=317 | mean (SD) | — |
| RHINE | faricimab 6 mg PTI/T&E | baseline | age | 61.6 (10.1) | years | n=319 | mean (SD) | — |
| RHINE | faricimab 6 mg PTI/T&E | baseline | male | 199 (62%) | patients | n=319 | n (%) | — |
| RHINE | faricimab 6 mg PTI/T&E | baseline | HbA1c | 7.7 (1.2) | % | n=319 | mean (SD) | — |
| RHINE | faricimab 6 mg PTI/T&E | baseline | BCVA | 62.5 (9.3) | ETDRS letters | n=319 | mean (SD) | — |
| RHINE | faricimab 6 mg PTI/T&E | baseline | CST | 471.3 (127.0) | µm | n=319 | mean (SD) | — |
| RHINE | aflibercept 2 mg q8w | baseline | age | 62.3 (10.1) | years | n=315 | mean (SD) | — |
| RHINE | aflibercept 2 mg q8w | baseline | male | 186 (59%) | patients | n=315 | n (%) | — |
| RHINE | aflibercept 2 mg q8w | baseline | HbA1c | 7.7 (1.2) | % | n=315 | mean (SD) | — |
| RHINE | aflibercept 2 mg q8w | baseline | BCVA | 62.1 (9.4) | ETDRS letters | n=315 | mean (SD) | — |
| RHINE | aflibercept 2 mg q8w | baseline | CST | 477.3 (129.4) | µm | n=315 | mean (SD) | — |
| YOSEMITE | Aflibercept 2 mg Q8W | Baseline | DMO duration | 17.5 (27.6) | months | n=312 | mean (SD) | — |
| RHINE | Aflibercept 2 mg Q8W | Baseline | DMO duration | 20.3 (37.1) | months | n=315 | mean (SD) | — |
| PHOTON | Aflibercept 2 mg Q8W | Baseline | Diabetes duration | 15.9 (10.0) | years | n=167 | mean (SD) | DMO duration not reported |
| YOSEMITE/RHINE | All relevant arms | Assessments | OCT platform/boundary | Spectralis, Cirrus or Topcon; ILM to Bruch’s membrane | — | — | definition | CST |
| PHOTON | All relevant arms | Assessments | OCT platform/boundary | SD-OCT; ILM to RPE; platform distribution not reported | — | — | definition | CRT |
| YOSEMITE | Aflibercept 2 mg Q8W | Baseline | Region | USA/Canada 54%; Asia 6%; other 40% | % | n=312 | n (%) | — |
| RHINE | Aflibercept 2 mg Q8W | Baseline | Region | USA/Canada 35%; Asia 8%; other 57% | % | n=315 | n (%) | — |
| PHOTON | Aflibercept 2 mg Q8W | Baseline | Region | Not reported by arm | — | n=167 | not reported | Seven countries overall |

Values were checked against the published trial baseline tables and were used only to assess cross-trial comparability.

**Supplementary Table S6. Complete conditional durability-scenario contrasts**

| **Scenario** | **Week** | **Outcome** | **Median** | **95% UI** | **Unit** | **P(Δ>0)** | **Outer draws** |
| --- | --- | --- | --- | --- | --- | --- | --- |
| **D1 reported-evidence reference** | 52 | Difference in active-visit fraction | +3.24 | +1.73 to +4.85 | pp | 100.0% | 500 |
| **D1 reported-evidence reference** | 52 | Difference unable to maintain ≥7 weeks | +7.23 | +2.30 to +13.47 | pp | 99.6% | 500 |
| **D1 reported-evidence reference** | 52 | Mean injection difference | +0.71 | +0.33 to +1.17 | injections | 100.0% | 500 |
| **D1 reported-evidence reference** | 52 | Difference in proportion at Q12W or longer | -17.23 | -26.97 to -8.43 | pp | 0.0% | 500 |
| **D1 reported-evidence reference** | 52 | Difference in proportion at Q16W or longer | -28.67 | -40.80 to -17.03 | pp | 0.0% | 500 |
| **D1 reported-evidence reference** | 52 | Difference with any interval shortening | +15.80 | +8.80 to +23.50 | pp | 100.0% | 500 |
| **D1 reported-evidence reference** | 104 | Difference in active-visit fraction | +3.02 | +0.53 to +5.54 | pp | 99.0% | 500 |
| **D1 reported-evidence reference** | 104 | Difference unable to maintain ≥7 weeks | +2.47 | -3.63 to +9.50 | pp | 79.0% | 500 |
| **D1 reported-evidence reference** | 104 | Mean injection difference | +1.66 | +0.84 to +2.47 | injections | 100.0% | 500 |
| **D1 reported-evidence reference** | 104 | Difference in proportion at Q12W or longer | -10.10 | -18.83 to +0.14 | pp | 2.6% | 500 |
| **D1 reported-evidence reference** | 104 | Difference in proportion at Q16W or longer | -16.80 | -27.40 to -3.62 | pp | 0.4% | 500 |
| **D1 reported-evidence reference** | 104 | Difference with any interval shortening | +21.00 | +8.50 to +34.10 | pp | 99.8% | 500 |
| **D2 exclude assigned extended intervals** | 52 | Difference in active-visit fraction | +3.31 | +1.75 to +5.09 | pp | 100.0% | 500 |
| **D2 exclude assigned extended intervals** | 52 | Difference unable to maintain ≥7 weeks | +7.53 | +2.10 to +13.23 | pp | 99.4% | 500 |
| **D2 exclude assigned extended intervals** | 52 | Mean injection difference | +0.73 | +0.31 to +1.16 | injections | 100.0% | 500 |
| **D2 exclude assigned extended intervals** | 52 | Difference in proportion at Q12W or longer | -17.57 | -26.20 to -7.83 | pp | 0.0% | 500 |
| **D2 exclude assigned extended intervals** | 52 | Difference in proportion at Q16W or longer | -29.80 | -42.41 to -16.86 | pp | 0.0% | 500 |
| **D2 exclude assigned extended intervals** | 52 | Difference with any interval shortening | +15.93 | +8.60 to +24.04 | pp | 100.0% | 500 |
| **D2 exclude assigned extended intervals** | 104 | Difference in active-visit fraction | +3.29 | +0.87 to +5.60 | pp | 99.8% | 500 |
| **D2 exclude assigned extended intervals** | 104 | Difference unable to maintain ≥7 weeks | +3.00 | -2.88 to +9.47 | pp | 86.2% | 500 |
| **D2 exclude assigned extended intervals** | 104 | Mean injection difference | +1.71 | +0.85 to +2.49 | injections | 100.0% | 500 |
| **D2 exclude assigned extended intervals** | 104 | Difference in proportion at Q12W or longer | -10.20 | -17.67 to -1.05 | pp | 1.4% | 500 |
| **D2 exclude assigned extended intervals** | 104 | Difference in proportion at Q16W or longer | -18.27 | -28.74 to -6.53 | pp | 0.0% | 500 |
| **D2 exclude assigned extended intervals** | 104 | Difference with any interval shortening | +22.40 | +8.23 to +34.53 | pp | 100.0% | 500 |
| **D3 downweight aflibercept maintained** | 52 | Difference in active-visit fraction | +2.72 | +0.74 to +4.46 | pp | 100.0% | 500 |
| **D3 downweight aflibercept maintained** | 52 | Difference unable to maintain ≥7 weeks | +6.73 | +1.17 to +13.33 | pp | 98.6% | 500 |
| **D3 downweight aflibercept maintained** | 52 | Mean injection difference | +0.64 | +0.21 to +1.13 | injections | 99.8% | 500 |
| **D3 downweight aflibercept maintained** | 52 | Difference in proportion at Q12W or longer | -14.87 | -25.43 to -4.28 | pp | 0.2% | 500 |
| **D3 downweight aflibercept maintained** | 52 | Difference in proportion at Q16W or longer | -25.20 | -38.81 to -9.66 | pp | 0.0% | 500 |
| **D3 downweight aflibercept maintained** | 52 | Difference with any interval shortening | +13.27 | +4.20 to +21.33 | pp | 100.0% | 500 |
| **D3 downweight aflibercept maintained** | 104 | Difference in active-visit fraction | +2.45 | -0.60 to +5.18 | pp | 94.2% | 500 |
| **D3 downweight aflibercept maintained** | 104 | Difference unable to maintain ≥7 weeks | +2.53 | -3.27 to +9.57 | pp | 80.6% | 500 |
| **D3 downweight aflibercept maintained** | 104 | Mean injection difference | +1.47 | +0.51 to +2.51 | injections | 100.0% | 500 |
| **D3 downweight aflibercept maintained** | 104 | Difference in proportion at Q12W or longer | -7.87 | -18.00 to +2.21 | pp | 5.2% | 500 |
| **D3 downweight aflibercept maintained** | 104 | Difference in proportion at Q16W or longer | -14.73 | -27.77 to +0.40 | pp | 3.0% | 500 |
| **D3 downweight aflibercept maintained** | 104 | Difference with any interval shortening | +17.27 | +2.13 to +32.33 | pp | 98.0% | 500 |
| **D4 downweight faricimab achieved** | 52 | Difference in active-visit fraction | +3.43 | +1.63 to +5.58 | pp | 100.0% | 500 |
| **D4 downweight faricimab achieved** | 52 | Difference unable to maintain ≥7 weeks | +6.47 | +0.66 to +13.27 | pp | 97.8% | 500 |
| **D4 downweight faricimab achieved** | 52 | Mean injection difference | +0.68 | +0.22 to +1.17 | injections | 99.8% | 500 |
| **D4 downweight faricimab achieved** | 52 | Difference in proportion at Q12W or longer | -17.57 | -29.24 to -7.40 | pp | 0.0% | 500 |
| **D4 downweight faricimab achieved** | 52 | Difference in proportion at Q16W or longer | -29.83 | -43.94 to -15.43 | pp | 0.0% | 500 |
| **D4 downweight faricimab achieved** | 52 | Difference with any interval shortening | +16.63 | +8.16 to +25.91 | pp | 100.0% | 500 |
| **D4 downweight faricimab achieved** | 104 | Difference in active-visit fraction | +3.66 | +0.89 to +6.06 | pp | 99.6% | 500 |
| **D4 downweight faricimab achieved** | 104 | Difference unable to maintain ≥7 weeks | +2.53 | -3.77 to +9.20 | pp | 84.2% | 500 |
| **D4 downweight faricimab achieved** | 104 | Mean injection difference | +1.65 | +0.71 to +2.64 | injections | 100.0% | 500 |
| **D4 downweight faricimab achieved** | 104 | Difference in proportion at Q12W or longer | -11.00 | -20.80 to -2.00 | pp | 0.8% | 500 |
| **D4 downweight faricimab achieved** | 104 | Difference in proportion at Q16W or longer | -20.23 | -31.20 to -6.98 | pp | 0.0% | 500 |
| **D4 downweight faricimab achieved** | 104 | Difference with any interval shortening | +23.67 | +9.00 to +36.37 | pp | 99.8% | 500 |
| **D5 one component** | 52 | Difference in active-visit fraction | +2.91 | +0.99 to +4.61 | pp | 99.8% | 500 |
| **D5 one component** | 52 | Difference unable to maintain ≥7 weeks | +5.13 | +2.23 to +9.03 | pp | 100.0% | 500 |
| **D5 one component** | 52 | Mean injection difference | +0.61 | +0.31 to +1.02 | injections | 100.0% | 500 |
| **D5 one component** | 52 | Difference in proportion at Q12W or longer | -17.30 | -26.54 to -9.03 | pp | 0.0% | 500 |
| **D5 one component** | 52 | Difference in proportion at Q16W or longer | -25.67 | -41.34 to -8.76 | pp | 0.4% | 500 |
| **D5 one component** | 52 | Difference with any interval shortening | +14.40 | +5.33 to +22.37 | pp | 99.8% | 500 |
| **D5 one component** | 104 | Difference in active-visit fraction | +1.75 | -1.49 to +4.62 | pp | 83.4% | 500 |
| **D5 one component** | 104 | Difference unable to maintain ≥7 weeks | +2.47 | -0.03 to +5.50 | pp | 96.8% | 500 |
| **D5 one component** | 104 | Mean injection difference | +1.41 | +0.77 to +2.19 | injections | 100.0% | 500 |
| **D5 one component** | 104 | Difference in proportion at Q12W or longer | -9.27 | -17.57 to +0.40 | pp | 3.4% | 500 |
| **D5 one component** | 104 | Difference in proportion at Q16W or longer | -13.00 | -30.48 to +6.10 | pp | 8.0% | 500 |
| **D5 one component** | 104 | Difference with any interval shortening | +12.00 | -5.01 to +27.83 | pp | 92.2% | 500 |
| **D6 three components** | 52 | Difference in active-visit fraction | +2.61 | +0.97 to +4.33 | pp | 100.0% | 500 |
| **D6 three components** | 52 | Difference unable to maintain ≥7 weeks | +5.33 | +1.46 to +10.10 | pp | 99.8% | 500 |
| **D6 three components** | 52 | Mean injection difference | +0.58 | +0.21 to +0.98 | injections | 100.0% | 500 |
| **D6 three components** | 52 | Difference in proportion at Q12W or longer | -15.17 | -25.60 to -5.60 | pp | 0.0% | 500 |
| **D6 three components** | 52 | Difference in proportion at Q16W or longer | -22.37 | -35.50 to -9.10 | pp | 0.0% | 500 |
| **D6 three components** | 52 | Difference with any interval shortening | +12.80 | +5.29 to +20.74 | pp | 100.0% | 500 |
| **D6 three components** | 104 | Difference in active-visit fraction | +2.39 | -0.10 to +4.78 | pp | 96.8% | 500 |
| **D6 three components** | 104 | Difference unable to maintain ≥7 weeks | +3.13 | -0.73 to +7.13 | pp | 94.4% | 500 |
| **D6 three components** | 104 | Mean injection difference | +1.38 | +0.67 to +2.13 | injections | 100.0% | 500 |
| **D6 three components** | 104 | Difference in proportion at Q12W or longer | -9.97 | -17.94 to -1.03 | pp | 1.6% | 500 |
| **D6 three components** | 104 | Difference in proportion at Q16W or longer | -14.77 | -26.80 to -0.69 | pp | 2.2% | 500 |
| **D6 three components** | 104 | Difference with any interval shortening | +15.70 | +1.53 to +27.94 | pp | 98.6% | 500 |
| **D7 stricter thresholds** | 52 | Difference in active-visit fraction | +3.17 | +1.45 to +5.14 | pp | 100.0% | 500 |
| **D7 stricter thresholds** | 52 | Difference unable to maintain ≥7 weeks | +9.60 | +3.39 to +17.17 | pp | 99.8% | 500 |
| **D7 stricter thresholds** | 52 | Mean injection difference | +0.88 | +0.44 to +1.41 | injections | 100.0% | 500 |
| **D7 stricter thresholds** | 52 | Difference in proportion at Q12W or longer | -19.20 | -28.98 to -10.16 | pp | 0.0% | 500 |
| **D7 stricter thresholds** | 52 | Difference in proportion at Q16W or longer | -31.77 | -46.71 to -17.20 | pp | 0.0% | 500 |
| **D7 stricter thresholds** | 52 | Difference with any interval shortening | +15.73 | +7.93 to +24.53 | pp | 100.0% | 500 |
| **D7 stricter thresholds** | 104 | Difference in active-visit fraction | +2.90 | +0.35 to +6.06 | pp | 98.6% | 500 |
| **D7 stricter thresholds** | 104 | Difference unable to maintain ≥7 weeks | +3.93 | -3.90 to +12.70 | pp | 84.6% | 500 |
| **D7 stricter thresholds** | 104 | Mean injection difference | +2.04 | +1.13 to +3.02 | injections | 100.0% | 500 |
| **D7 stricter thresholds** | 104 | Difference in proportion at Q12W or longer | -11.97 | -21.00 to -1.46 | pp | 0.6% | 500 |
| **D7 stricter thresholds** | 104 | Difference in proportion at Q16W or longer | -18.17 | -31.07 to -5.25 | pp | 0.4% | 500 |
| **D7 stricter thresholds** | 104 | Difference with any interval shortening | +20.83 | +7.00 to +37.71 | pp | 99.8% | 500 |
| **D8 permissive thresholds** | 52 | Difference in active-visit fraction | +3.09 | +1.83 to +4.57 | pp | 100.0% | 500 |
| **D8 permissive thresholds** | 52 | Difference unable to maintain ≥7 weeks | +5.13 | +1.40 to +10.70 | pp | 99.4% | 500 |
| **D8 permissive thresholds** | 52 | Mean injection difference | +0.55 | +0.23 to +0.95 | injections | 100.0% | 500 |
| **D8 permissive thresholds** | 52 | Difference in proportion at Q12W or longer | -15.00 | -24.10 to -6.67 | pp | 0.0% | 500 |
| **D8 permissive thresholds** | 52 | Difference in proportion at Q16W or longer | -26.03 | -36.80 to -15.26 | pp | 0.0% | 500 |
| **D8 permissive thresholds** | 52 | Difference with any interval shortening | +14.70 | +8.67 to +21.70 | pp | 100.0% | 500 |
| **D8 permissive thresholds** | 104 | Difference in active-visit fraction | +2.90 | +0.76 to +5.09 | pp | 99.8% | 500 |
| **D8 permissive thresholds** | 104 | Difference unable to maintain ≥7 weeks | +1.47 | -3.14 to +7.50 | pp | 74.8% | 500 |
| **D8 permissive thresholds** | 104 | Mean injection difference | +1.32 | +0.63 to +2.03 | injections | 100.0% | 500 |
| **D8 permissive thresholds** | 104 | Difference in proportion at Q12W or longer | -8.17 | -16.43 to +1.40 | pp | 5.0% | 500 |
| **D8 permissive thresholds** | 104 | Difference in proportion at Q16W or longer | -15.13 | -25.20 to -3.23 | pp | 0.6% | 500 |
| **D8 permissive thresholds** | 104 | Difference with any interval shortening | +19.60 | +8.09 to +30.14 | pp | 100.0% | 500 |
| **D9 low visit noise** | 52 | Difference in active-visit fraction | +1.18 | +0.50 to +1.89 | pp | 100.0% | 500 |
| **D9 low visit noise** | 52 | Difference unable to maintain ≥7 weeks | +8.10 | +2.10 to +15.40 | pp | 99.4% | 500 |
| **D9 low visit noise** | 52 | Mean injection difference | +0.76 | +0.33 to +1.23 | injections | 100.0% | 500 |
| **D9 low visit noise** | 52 | Difference in proportion at Q12W or longer | -17.67 | -27.43 to -7.60 | pp | 0.0% | 500 |
| **D9 low visit noise** | 52 | Difference in proportion at Q16W or longer | -29.40 | -41.40 to -16.96 | pp | 0.0% | 500 |
| **D9 low visit noise** | 52 | Difference with any interval shortening | +5.73 | +2.67 to +9.17 | pp | 100.0% | 500 |
| **D9 low visit noise** | 104 | Difference in active-visit fraction | +0.39 | -1.68 to +2.25 | pp | 66.2% | 500 |
| **D9 low visit noise** | 104 | Difference unable to maintain ≥7 weeks | +2.67 | -4.44 to +11.00 | pp | 75.6% | 500 |
| **D9 low visit noise** | 104 | Mean injection difference | +1.73 | +0.88 to +2.65 | injections | 100.0% | 500 |
| **D9 low visit noise** | 104 | Difference in proportion at Q12W or longer | -10.90 | -20.47 to -0.06 | pp | 2.6% | 500 |
| **D9 low visit noise** | 104 | Difference in proportion at Q16W or longer | -16.50 | -27.77 to -2.39 | pp | 0.8% | 500 |
| **D9 low visit noise** | 104 | Difference with any interval shortening | +5.37 | -6.38 to +15.57 | pp | 81.4% | 500 |
| **D10 high visit noise** | 52 | Difference in active-visit fraction | +4.71 | +2.61 to +7.15 | pp | 100.0% | 500 |
| **D10 high visit noise** | 52 | Difference unable to maintain ≥7 weeks | +6.37 | +2.20 to +11.43 | pp | 99.8% | 500 |
| **D10 high visit noise** | 52 | Mean injection difference | +0.68 | +0.37 to +1.09 | injections | 100.0% | 500 |
| **D10 high visit noise** | 52 | Difference in proportion at Q12W or longer | -16.33 | -24.90 to -8.59 | pp | 0.0% | 500 |
| **D10 high visit noise** | 52 | Difference in proportion at Q16W or longer | -26.93 | -39.87 to -16.00 | pp | 0.0% | 500 |
| **D10 high visit noise** | 52 | Difference with any interval shortening | +21.33 | +12.09 to +31.90 | pp | 100.0% | 500 |
| **D10 high visit noise** | 104 | Difference in active-visit fraction | +5.07 | +1.99 to +8.51 | pp | 100.0% | 500 |
| **D10 high visit noise** | 104 | Difference unable to maintain ≥7 weeks | +2.40 | -2.10 to +8.43 | pp | 84.0% | 500 |
| **D10 high visit noise** | 104 | Mean injection difference | +1.58 | +0.88 to +2.31 | injections | 100.0% | 500 |
| **D10 high visit noise** | 104 | Difference in proportion at Q12W or longer | -9.47 | -17.63 to -0.33 | pp | 1.6% | 500 |
| **D10 high visit noise** | 104 | Difference in proportion at Q16W or longer | -16.60 | -26.84 to -5.55 | pp | 0.2% | 500 |
| **D10 high visit noise** | 104 | Difference with any interval shortening | +24.97 | +8.86 to +41.36 | pp | 100.0% | 500 |
| **D11 equal durability (null)** | 52 | Difference in active-visit fraction | +0.00 | +0.00 to +0.00 | pp | 0.0% | 500 |
| **D11 equal durability (null)** | 52 | Difference unable to maintain ≥7 weeks | +0.00 | +0.00 to +0.00 | pp | 0.0% | 500 |
| **D11 equal durability (null)** | 52 | Mean injection difference | +0.00 | +0.00 to +0.00 | injections | 0.0% | 500 |
| **D11 equal durability (null)** | 52 | Difference in proportion at Q12W or longer | +0.00 | +0.00 to +0.00 | pp | 0.0% | 500 |
| **D11 equal durability (null)** | 52 | Difference in proportion at Q16W or longer | +0.00 | +0.00 to +0.00 | pp | 0.0% | 500 |
| **D11 equal durability (null)** | 52 | Difference with any interval shortening | +0.00 | +0.00 to +0.00 | pp | 0.0% | 500 |
| **D11 equal durability (null)** | 104 | Difference in active-visit fraction | +0.00 | +0.00 to +0.00 | pp | 0.0% | 500 |
| **D11 equal durability (null)** | 104 | Difference unable to maintain ≥7 weeks | +0.00 | +0.00 to +0.00 | pp | 0.0% | 500 |
| **D11 equal durability (null)** | 104 | Mean injection difference | +0.00 | +0.00 to +0.00 | injections | 0.0% | 500 |
| **D11 equal durability (null)** | 104 | Difference in proportion at Q12W or longer | +0.00 | +0.00 to +0.00 | pp | 0.0% | 500 |
| **D11 equal durability (null)** | 104 | Difference in proportion at Q16W or longer | +0.00 | +0.00 to +0.00 | pp | 0.0% | 500 |
| **D11 equal durability (null)** | 104 | Difference with any interval shortening | +0.00 | +0.00 to +0.00 | pp | 0.0% | 500 |

*All contrasts are faricimab minus aflibercept 8 mg. For interval outcomes, negative values indicate a lower modelled proportion with faricimab. D11 is a null structural check, not evidence of equal durability.*

**Supplementary Table S7. Calibration-error-expanded burden sensitivity ranges**

| **Week** | **Outcome** | **Median** | **Nominal 95% UI** | **Empirical bound** | **Empirical-adjusted interval** | **Tolerance bound** | **Tolerance-adjusted interval** | **Unit** |
| --- | --- | --- | --- | --- | --- | --- | --- | --- |
| 52 | Difference in active-visit fraction | +3.24 | +1.73 to +4.85 | 9.85 | -8.12 to +14.70 | 20.00 | -18.27 to +24.85 | pp |
| 52 | Difference unable to maintain ≥7 weeks | +7.23 | +2.30 to +13.47 | 9.85 | -7.55 to +23.32 | 20.00 | -17.70 to +33.47 | pp |
| 52 | Mean injection difference | +0.71 | +0.33 to +1.17 | 1.26 | -0.93 to +2.42 | 2.00 | -1.67 to +3.17 | injections |
| 52 | Difference in proportion at Q12W or longer | -17.23 | -26.97 to -8.43 | 3.47 | -30.44 to -4.96 | 20.00 | -46.97 to +11.57 | pp |
| 52 | Difference in proportion at Q16W or longer | -28.67 | -40.80 to -17.03 | 9.85 | -50.65 to -7.18 | 20.00 | -60.80 to +2.97 | pp |
| 52 | Difference with any interval shortening | +15.80 | +8.80 to +23.50 | 9.85 | −1.05 to +33.35 | 20.00 | −11.20 to +43.50 | pp |
| 104 | Difference in active-visit fraction | +3.02 | +0.53 to +5.54 | 9.85 | -9.32 to +15.39 | 20.00 | -19.47 to +25.54 | pp |
| 104 | Difference unable to maintain ≥7 weeks | +2.47 | -3.63 to +9.50 | 9.85 | -13.48 to +19.35 | 20.00 | -23.64 to +29.50 | pp |
| 104 | Mean injection difference | +1.66 | +0.84 to +2.47 | 1.26 | -0.41 to +3.73 | 2.00 | -1.16 to +4.47 | injections |
| 104 | Difference in proportion at Q12W or longer | -10.10 | -18.83 to +0.14 | 3.47 | -22.31 to +3.61 | 20.00 | -38.83 to +20.14 | pp |
| 104 | Difference in proportion at Q16W or longer | -16.80 | -27.40 to -3.62 | 9.85 | -37.25 to +6.23 | 20.00 | -47.40 to +16.38 | pp |
| 104 | Difference with any interval shortening | +21.00 | +8.50 to +34.10 | 9.85 | −1.35 to +43.95 | 20.00 | −11.50 to +54.10 | pp |

The empirical sensitivity range expands the nominal uncertainty interval by the combined maximum absolute replication errors for the two treatments. This is a deterministic sensitivity bound, not a posterior uncertainty interval; using the engineering tolerances produces a more conservative range.

**Supplementary Table S8. Selected descriptive ocular safety outcomes**

| **Trial** | **Arm** | **Timepoint** | **Variable** | **Value** | **Denominator** | **Statistic** | **Verification** |
| --- | --- | --- | --- | --- | --- | --- | --- |
| PHOTON | aflibercept 2 mg q8w | week 48 | intraocular inflammation | 1 (1%) | n=167 | n (%) | Verified against PHOTON week-48 safety table; safety set denominator |
| PHOTON | aflibercept 8 mg q12w | week 48 | intraocular inflammation | 4 (1%) | n=328 | n (%) | Verified against PHOTON week-48 safety table; safety set denominator |
| PHOTON | aflibercept 8 mg q16w | week 48 | intraocular inflammation | 0 | n=163 | n (%) | Verified against PHOTON week-48 safety table; safety set denominator |
| PHOTON | aflibercept 2 mg q8w | week 96 | treatment-related ocular TEAE | 3 (1.8%) | n=167 | n (%) | Verified against PHOTON week-96 safety table; descriptive, not poolable with week-48 table |
| PHOTON | aflibercept 2 mg q8w | week 96 | intraocular inflammation | 2 (1.2%) | n=167 | n (%) | Verified against PHOTON week-96 safety table; descriptive, not poolable with week-48 table |
| PHOTON | aflibercept 8 mg q12w | week 96 | treatment-related ocular TEAE | 8 (2.4%) | n=328 | n (%) | Verified against PHOTON week-96 safety table; descriptive, not poolable with week-48 table |
| PHOTON | aflibercept 8 mg q12w | week 96 | intraocular inflammation | 5 (1.5%) | n=328 | n (%) | Verified against PHOTON week-96 safety table; descriptive, not poolable with week-48 table |
| PHOTON | aflibercept 8 mg q16w | week 96 | treatment-related ocular TEAE | 1 (0.6%) | n=163 | n (%) | Verified against PHOTON week-96 safety table; descriptive, not poolable with week-48 table |
| PHOTON | aflibercept 8 mg q16w | week 96 | intraocular inflammation | 1 (0.6%) | n=163 | n (%) | Verified against PHOTON week-96 safety table; descriptive, not poolable with week-48 table |
| YOSEMITE | faricimab 6 mg q8w | week 56 | intraocular inflammation | 5 (2%) | n=313 | n (%) | Verified against YOSEMITE/RHINE year-1 safety table; safety population |
| YOSEMITE | faricimab 6 mg q8w | week 56 | endophthalmitis | 0 | n=313 | n (%) | Verified against YOSEMITE/RHINE year-1 safety table; safety population |
| YOSEMITE | faricimab 6 mg q8w | week 56 | retinal vasculitis | 0 | n=313 | n (%) | Verified against YOSEMITE/RHINE year-1 safety table; safety population |
| YOSEMITE | faricimab 6 mg PTI/T&E | week 56 | intraocular inflammation | 7 (2%) | n=313 | n (%) | Verified against YOSEMITE/RHINE year-1 safety table; safety population |
| YOSEMITE | faricimab 6 mg PTI/T&E | week 56 | endophthalmitis | 2 (1%) | n=313 | n (%) | Verified against YOSEMITE/RHINE year-1 safety table; safety population |
| YOSEMITE | faricimab 6 mg PTI/T&E | week 56 | retinal vasculitis | 0 | n=313 | n (%) | Verified against YOSEMITE/RHINE year-1 safety table; safety population |
| YOSEMITE | aflibercept 2 mg q8w | week 56 | intraocular inflammation | 3 (1%) | n=311 | n (%) | Verified against YOSEMITE/RHINE year-1 safety table; safety population |
| YOSEMITE | aflibercept 2 mg q8w | week 56 | endophthalmitis | 0 | n=311 | n (%) | Verified against YOSEMITE/RHINE year-1 safety table; safety population |
| YOSEMITE | aflibercept 2 mg q8w | week 56 | retinal vasculitis | 0 | n=311 | n (%) | Verified against YOSEMITE/RHINE year-1 safety table; safety population |
| RHINE | faricimab 6 mg q8w | week 56 | intraocular inflammation | 3 (1%) | n=317 | n (%) | Verified against YOSEMITE/RHINE year-1 safety table; safety population |
| RHINE | faricimab 6 mg q8w | week 56 | endophthalmitis | 2 (1%) | n=317 | n (%) | Verified against YOSEMITE/RHINE year-1 safety table; safety population |
| RHINE | faricimab 6 mg q8w | week 56 | retinal vasculitis | 0 | n=317 | n (%) | Verified against YOSEMITE/RHINE year-1 safety table; safety population |
| RHINE | faricimab 6 mg PTI/T&E | week 56 | intraocular inflammation | 2 (1%) | n=319 | n (%) | Verified against YOSEMITE/RHINE year-1 safety table; safety population |
| RHINE | faricimab 6 mg PTI/T&E | week 56 | endophthalmitis | 0 | n=319 | n (%) | Verified against YOSEMITE/RHINE year-1 safety table; safety population |
| RHINE | faricimab 6 mg PTI/T&E | week 56 | retinal vasculitis | 0 | n=319 | n (%) | Verified against YOSEMITE/RHINE year-1 safety table; safety population |
| RHINE | aflibercept 2 mg q8w | week 56 | intraocular inflammation | 1 (<1%) | n=314 | n (%) | Verified against YOSEMITE/RHINE year-1 safety table; safety population |
| RHINE | aflibercept 2 mg q8w | week 56 | endophthalmitis | 1 (<1%) | n=314 | n (%) | Verified against YOSEMITE/RHINE year-1 safety table; safety population |
| RHINE | aflibercept 2 mg q8w | week 56 | retinal vasculitis | 0 | n=314 | n (%) | Verified against YOSEMITE/RHINE year-1 safety table; safety population |
| YOSEMITE | faricimab 6 mg q8w | study end/week 100 | intraocular inflammation | 6 (1.9%) | n=313 | n (%) | Verified against YOSEMITE/RHINE study-end safety table; safety population |
| YOSEMITE | faricimab 6 mg q8w | study end/week 100 | endophthalmitis | 0 | n=313 | n (%) | Verified against YOSEMITE/RHINE study-end safety table; safety population |
| YOSEMITE | faricimab 6 mg q8w | study end/week 100 | retinal vasculitis | 0 | n=313 | n (%) | Verified against YOSEMITE/RHINE study-end safety table; safety population |
| YOSEMITE | faricimab 6 mg PTI/T&E | study end/week 100 | intraocular inflammation | 7 (2.2%) | n=313 | n (%) | Verified against YOSEMITE/RHINE study-end safety table; safety population |
| YOSEMITE | faricimab 6 mg PTI/T&E | study end/week 100 | endophthalmitis | 3 (1.0%) | n=313 | n (%) | Verified against YOSEMITE/RHINE study-end safety table; safety population |
| YOSEMITE | faricimab 6 mg PTI/T&E | study end/week 100 | retinal vasculitis | 0 | n=313 | n (%) | Verified against YOSEMITE/RHINE study-end safety table; safety population |
| YOSEMITE | aflibercept 2 mg q8w | study end/week 100 | intraocular inflammation | 5 (1.6%) | n=311 | n (%) | Verified against YOSEMITE/RHINE study-end safety table; safety population |
| YOSEMITE | aflibercept 2 mg q8w | study end/week 100 | endophthalmitis | 0 | n=311 | n (%) | Verified against YOSEMITE/RHINE study-end safety table; safety population |
| YOSEMITE | aflibercept 2 mg q8w | study end/week 100 | retinal vasculitis | 0 | n=311 | n (%) | Verified against YOSEMITE/RHINE study-end safety table; safety population |
| RHINE | faricimab 6 mg q8w | study end/week 100 | intraocular inflammation | 3 (0.9%) | n=317 | n (%) | Verified against YOSEMITE/RHINE study-end safety table; safety population |
| RHINE | faricimab 6 mg q8w | study end/week 100 | endophthalmitis | 2 (0.6%) | n=317 | n (%) | Verified against YOSEMITE/RHINE study-end safety table; safety population |
| RHINE | faricimab 6 mg q8w | study end/week 100 | retinal vasculitis | 0 | n=317 | n (%) | Verified against YOSEMITE/RHINE study-end safety table; safety population |
| RHINE | faricimab 6 mg PTI/T&E | study end/week 100 | intraocular inflammation | 4 (1.3%) | n=319 | n (%) | Verified against YOSEMITE/RHINE study-end safety table; safety population |
| RHINE | faricimab 6 mg PTI/T&E | study end/week 100 | endophthalmitis | 1 (0.3%) | n=319 | n (%) | Verified against YOSEMITE/RHINE study-end safety table; safety population |
| RHINE | faricimab 6 mg PTI/T&E | study end/week 100 | retinal vasculitis | 0 | n=319 | n (%) | Verified against YOSEMITE/RHINE study-end safety table; safety population |
| RHINE | aflibercept 2 mg q8w | study end/week 100 | intraocular inflammation | 2 (0.6%) | n=314 | n (%) | Verified against YOSEMITE/RHINE study-end safety table; safety population |
| RHINE | aflibercept 2 mg q8w | study end/week 100 | endophthalmitis | 1 (0.3%) | n=314 | n (%) | Verified against YOSEMITE/RHINE study-end safety table; safety population |
| RHINE | aflibercept 2 mg q8w | study end/week 100 | retinal vasculitis | 0 | n=314 | n (%) | Verified against YOSEMITE/RHINE study-end safety table; safety population |

*Safety is described by source programme only. No cross-trial statistical comparison was performed.*

**Appendix S1. Statistical model specification**

**S1.1 Analytical separation**

Let Δe,t denote the faricimab-minus-aflibercept 8 mg aggregate efficacy contrast for endpoint e at time t. Let Δb,w denote a conditional microsimulation contrast for burden outcome b at week w. The model does not set Δe,t = f(dosing history), and no simulated injection, interval, adherence or shortening variable enters the efficacy likelihood. Conversely, efficacy draws do not determine scheduling events. The two outputs must therefore be interpreted separately, and protocol standardisation applies only to Δb,w.

**S1.2 Anchored aggregate efficacy likelihood**

For each endpoint and year, y ~ MVN(Xβ, Vρ + τ²I), where y contains within-trial contrasts against aflibercept 2 mg; X contains an intercept, a faricimab indicator and nominal regimen interval centred at 12 weeks; Vρ contains reported or reconstructed sampling variances and covariance for contrasts sharing a comparator; and τ is residual between-contrast heterogeneity. The faricimab coefficient is the anchored relative efficacy estimate at the model reference value corresponding to a nominal 12-week regimen label. This meta-regression adjustment does not recreate a common dosing history or protocol-standardise efficacy. The reference shared-comparator correlation was ρ=0.50.

The published week-12 matching-adjusted indirect comparison supplied means of +0.8 letters (95% credible interval −0.7 to +2.2) and +19.0 µm greater reduction (+3.2 to +35.0), after direction harmonisation. Standard deviations reconstructed from the published week-12 credible intervals were multiplied by 2 to weaken this overlapping information; year-2 prior standard deviations were 1.5 letters and 20 µm. E1 used weak zero-centred priors with standard deviations of 5 letters and 50 µm; E2 used sceptical zero-centred priors with standard deviations of 1 letter and 10 µm. E7–E10 omitted the interval covariate and week-12 prior and additionally omitted the named trial or regimen. Individual baseline efficacy distributions do not enter either estimand.

**S1.3 Conditional durability model**

The reference latent sustainable interval used a two-component normal mixture: D|C=L ~ Normal(μL,σ²), D|C=H ~ Normal(μH,σ²), and P(C=H)=π. Category thresholds mapped continuous durability to Q4W, Q8W, Q12W or Q16W. Transported likelihood contributions were power-discounted, but discounting was not treated as correction for directional protocol bias. D5 and D6 used one- and three-component structures; D2–D4 changed evidence interpretation; D7–D8 changed thresholds; D9–D10 changed visit noise; D11 imposed equal durability.

Year-1 parameters governed weeks 12–52. Between weeks 52 and 96, π was interpolated linearly on the logit scale, μ values on the natural scale and σ on the log scale. Year-2 parameters governed weeks 96–104. A persistent patient rank induced correlation over time. Because π could change, this did not define a fixed immutable subgroup.

**S1.4 Event clock**

The clock advances in integer weeks. Loading injections occur at weeks 0, 4 and 8. At week 12, a pre-dose assessment determines the next interval, and the fourth injection is administered. Subsequent treatment events occur at the scheduled interval. The primary model is therefore a three-loading-injection regimen; a distinct sensitivity scenario gives a fourth loading injection before extension.

**S1.5 Qualification and calibration**

Model qualification assessed reproducibility, covariance validity, posterior support and replication of original-regimen outcomes. Tolerances were one injection for injection-count targets, 10 percentage points for critical interval targets and 12 percentage points for exploratory extended-interval targets. Target classifications and tolerances were defined before the reported qualification assessment but were not prespecified a priori. Nominal comparative uncertainty intervals were also expanded using the combined maximum original-regimen replication errors across treatments. These expanded ranges are deterministic sensitivity bounds, not posterior uncertainty intervals.

**S1.6 Posterior reporting**

Posterior medians and 2.5th–97.5th percentile uncertainty intervals are reported. P(Δ>0) is used only when its direction is explicit. The virtual cohort size controls numerical integration error and is not an empirical sample size.

**Appendix S2. Computational reproducibility**

| **Item** | **Configuration/ result** |
| --- | --- |
| **R** | 4.6.1 (2026-06-24, ucrt) |
| **Reference efficacy draws** | 4,000 |
| **Draws per efficacy structural case** | 3,000 |
| **Outer draws per durability scenario** | 500 |
| **Virtual patients per arm** | 1,500 |
| **Warnings** | None recorded |
| **Critical qualification** | 13/13 passed |
| **All qualification targets** | 15/17 passed |
| **Code review** | No independent reviewer or double programming |
| **Integrity controls** | Automated tests; manifest; SHA-256 checksums; Exact reproduction of reported results |

Detailed execution instructions are provided in the archive’s README file. The versioned v5.4.2 Zenodo archive contains the model inputs, derived data, R code, configurations, automated tests, authoritative outputs, session information, manifest and checksums.

**Appendix S3. Evidence-search methods**

| **Element** | **Specification** |
| --- | --- |
| **Databases** | MEDLINE/PubMed; ClinicalTrials.gov |
| **Other sources** | Trial registries, journal supplements, conference abstracts/posters and backward citation searching |
| **Coverage** | Targeted searches updated to 30 June 2026 |
| **Core concepts** | diabetic macular oedema/edema; faricimab; aflibercept 8 mg; YOSEMITE; RHINE; PHOTON; BCVA; retinal thickness; interval; injections; durability; safety |
| **Inclusion** | Pivotal randomised or prespecified external cohorts reporting extractable aggregate efficacy, anatomy, burden, interval, qualification or safety data |
| **Exclusion** | No DMO subgroup; non-extractable outcomes; duplicate interim report superseded by a fuller source; commentary without primary data |
| **Use hierarchy** | Randomised within-trial contrasts for efficacy; pivotal interval data for conditional burden; observational studies for external plausibility only |
| **Extraction** | One author; source and transformation recorded; every model-critical value checked twice by the same author |
| Direct comparative context | Maccauro et al. (2025) and Menna et al. (2026) retained for context only; excluded from model inputs, calibration and validation |

The search identified two small retrospective direct comparisons (Maccauro et al., 2025; Menna et al., 2026). They were retained only to contextualise the evidence gap and excluded from model inputs, calibration and validation because of non-randomised treatment selection, short follow-up and protocol differences. A PRISMA claim is not made because this was a targeted model-input search rather than a systematic review of comparative clinical effects.

**Supplementary figure legends**

**Supplementary Figure S1**. Qualification of the treatment-burden model against 17 original-regimen targets. Targets T1–T13 were classified as critical and T14–T17 as exploratory. Values represent the absolute observed–predicted error relative to the specified tolerance.

**Supplementary Figure S2.** Sensitivity analyses of the anchored efficacy comparison for BCVA and retinal thickness at years 1 and 2. Points show posterior medians and 95% uncertainty intervals. E7–E10 omit the nominal-interval covariate, the week-12 prior and one specified trial or regimen.
